# Molecular Impact of Sublethal Spinetoram Exposure on Honeybee (*Apis mellifera*) Larval and Adult Transcriptomes

**DOI:** 10.3390/ijms252211923

**Published:** 2024-11-06

**Authors:** Bala Murali Krishna Vasamsetti, Juyeong Kim, Kyongmi Chon, Bo-Seon Kim, Chang-Young Yoon, Sojeong Hwang, Kyeong-Hun Park

**Affiliations:** Toxicity and Risk Assessment Division, Department of Agro-Food Safety and Crop Protection, National Institute of Agricultural Sciences, Rural Development Administration, Iseo-myeon, Wanju-gun 55365, Jeollabuk-do, Republic of Korea; vbmk84@gmail.com (B.M.K.V.); kjy.sara@gmail.com (J.K.); kbs9249@naver.com (B.-S.K.); evermoo2600@korea.kr (C.-Y.Y.); hsj102@korea.kr (S.H.); blueour@korea.kr (K.-H.P.)

**Keywords:** differentially expressed genes, gene ontology analysis, honeybee, KEGG pathway enrichment analysis, pesticide toxicity, spinetoram, transcriptome

## Abstract

Pesticide toxicity is a global concern for honeybee populations, and understanding these effects at the molecular level is critical. This study analyzed the transcriptome of honeybees at larval and adult stages after chronic exposure to a sublethal dose (0.0017 µg a.i./larva) of spinetoram (SPI) during the larval phase. Four groups were used: acetone-treated honeybee larvae (ATL), acetone-treated honeybee adults (ATAs), SPI-treated honeybee larvae (STL), and SPI-treated honeybee adults (STAs). In total, 5719 differentially expressed genes (DEGs) were identified for ATL vs. ATAs, 5754 for STL vs. STAs, 273 for ATL vs. STL, and 203 for ATAs vs. STAs (FC ≤ 1.5, *p* < 0.05). In response to SPI, 29 unique DEGs were identified in larvae and 42 in adults, with 23 overlapping between comparisons, suggesting genes linked to SPI toxicity. Gene ontology analysis showed that SPI affected metabolism-related genes in larvae and lipid-transport-associated genes in adults. KEGG pathway analysis revealed an enrichment of pathways predominantly associated with metabolism, hormone biosynthesis, and motor proteins in STL. The transcriptomic data were validated by qPCR. These findings demonstrated that SPI disrupts essential molecular processes, potentially harming honeybee development and behavior, underscoring the need for safer agricultural practices.

## 1. Introduction

Honeybees play a crucial role in ecosystems by pollinating various plant species and providing products, like honey and beeswax [1]. The global decline in honeybee populations has become a pressing issue, leading to extensive research to understand the factors contributing to this decline [2,3,4]. Among these, pesticide exposure is considered a significant threat [2,3,4]. Bees forage over large areas, often ingesting pesticides that impair their foraging behavior, navigation, and immune response, intensifying the effects of other stressors, like pathogens and habitat loss [2,5,6,7]. This combination of stressors can result in phenomena, like colony collapse disorder, and contribute to the broader decline of honeybee populations. Larvae are particularly vulnerable, with several studies showing that pesticide exposure increases larval mortality, reduces hatching rates, and causes deformities in wings and antennae [8,9].

Neonicotinoids are one of the most widely used pesticides worldwide; however, their off-target toxicity has raised concerns, resulting in several being banned in the European Union and restricted in the US [10]. This has led to the increased use of alternative pesticide families, including the spinosyn group [11]. Spinetoram (SPI), a member of this group, acts as a nicotinic acetylcholine receptor (nAChR) agonist and is effective against lepidopteran pests [12]. Spinosyns, such as spinosad, activate nAChRs in the insect nervous system, causing neural overexcitation, leading to muscle contractions, tremors, loss of coordination, neuromuscular fatigue, paralysis, and death, while also antagonizing desensitized nAChRs at lower doses [13,14]. Specifically, spinosad targets the nAChRα6 subunit, disrupting calcium signaling and triggering lysosomal dysfunction, oxidative stress through reactive oxygen species (ROS) production, mitochondrial stress, and eventually, cellular damage and neurodegeneration [15,16]. Notably, SPI has demonstrated high toxicity in the adult and larval stages of certain insects, comparable to neonicotinoids and organophosphates [17], highlighting the need for comprehensive nontarget toxicity assessments.

Several studies have documented the toxic effects of SPI on nontarget organisms, which include oxidative stress [12,18,19,20]. SPI is toxic to humans, causing cytotoxic effects in human liver cells via autophagy and oxidative DNA damage, as well as inhibiting HepG2 cell viability via DNA damage and mitochondria-associated apoptosis [18,19]. Mysid shrimp (*Americamysis bahia*) exposed to SPI exhibited sublethal effects, such as floating, lethargy, and erratic swimming, and chronic exposure affected their reproduction [12]. Eastern oysters (*Crassostrea virginica*) exposed to SPI showed a complete absence of shell growth [12]. SPI also caused significant changes in oxidative-stress-related indicators in zebrafish, including increased levels of ROS and malondialdehyde, as well as alterations in the activity of antioxidant enzymes, such as catalase and superoxide dismutase (SOD) [20]. SPI exhibits both direct and chronic toxic effects on bumblebees (*Bombus terrestris*), reducing mortality, reproductive success, and foraging behavior [21]. SPI also decreases the lifespan and parasitism capacity of whitefly wasp (*Encarsia formosa*) adults, impacting population growth [22]. In field trials, SPI reduced adult and larval emergence in South American fruit fly (*Anastrepha fraterculus*) and medfly (*Ceratitis capitata*) [17], while sublethal exposure accelerated development and increased fecundity in two-spotted spider mites (*Tetranychus urticae*), potentially leading to pest outbreaks [23].

According to the US Environmental Protection Agency (EPA), SPI is toxic to bees, especially when applied directly or when residues are present on flowering plants or weeds [24]. Consequently, refraining from using this product or not permitting it to drift onto flowering plants or weeds is recommended if bee activity is present in the treatment area [24]. Bumblebees exposed to sublethal doses of SPI showed a notable increase in SOD activity associated with a significant decrease in intestinal amylase activity [25]. Nancy Ostiguy et al. found that the maximum SPI residue detected in pollen was 645 µg/kg in 2011 in a nationwide study conducted in the US [26]. Honeybees demonstrated acute sensitivity to contact and oral exposure to SPI, with a dose-dependent increase in mortality [12]. The reported LD_50_ values were 0.024 µg/bee for the contact test and 0.14 µg/bee for the oral test, respectively [27]. Our previous study determined the acute and chronic LD_50_ values of SPI for honeybee larvae to be 0.026 and 0.017 μg a.i/larva, respectively, underscoring the compound’s significant toxicity and potential danger to bee populations [28].

Toxicity assessments of honeybees typically focus on evaluating mortality, minimal phenotype scoring (antenna and wing deformities), and behavioral changes [8,29]. Pesticides are chemically complex, and any discernible phenotypic changes in honeybees following pesticide exposure are likely the result of intricate molecular alterations [30]. Traditional methods for assessing the molecular toxicity of pesticides in honeybees, such as ELISA and qPCR, are often labor-intensive and provide limited insights into the molecular mechanisms of toxicity [28,31,32]. In contrast, next-generation whole-transcriptome sequencing is a powerful tool for revealing complex molecular changes and has been successfully applied to various organisms [33,34,35,36,37].

Therefore, the present study aimed to analyze global transcriptomic changes and subsequently perform functional enrichment analysis to profoundly comprehend the sublethal toxicity induced by SPI in honeybees at the molecular level. Using whole-transcriptome sequencing, we compared gene expression in the SPI-treated group with that in the acetone-treated control group. Differentially expressed genes (DEGs) were identified and validated using qRT-PCR, followed by gene ontology (GO) and Kyoto Encyclopedia of Genes and Genomes (KEGG) pathway enrichment analyses. This study provides molecular insights into the genes and pathways associated with SPI-induced toxicity in honeybees.

## 2. Results

### 2.1. Analysis of DEGs in Spinetoram-Exposed and Acetone-Exposed Honeybee Larvae and Adults

High-throughput paired-end transcriptome sequencing was performed on the following groups using the Illumina platform: acetone-treated honeybee larvae (ATL), acetone-treated honeybee adults (ATAs), SPI-treated honeybee larvae (STL), and SPI-treated honeybee adults (STAs). Sequencing analysis revealed that the three biological replicates of each sample had an average of 58.39 million reads for ATAs, 55.00 million reads for ATL, 57.34 million reads for STAs, and 60.11 million reads for STL (Table 1). After removing adapters and low-quality tags, an average of 57.03 million clean reads were achieved for ATAs, 53.56 million for ATL, 56.00 million for STAs, and 58.78 million for STL. The clean reads exhibited an average GC content of 38.06%, with a Q20 score of 98.71% and a Q30 score of 97.74%, respectively. The clean reads were aligned to the reference genome (*Apis mellifera*, Amel_HAv3.1, and NCBI_20180910) and showed an average mapping rate of over 97% across all sample groups.

**Figure 1:**
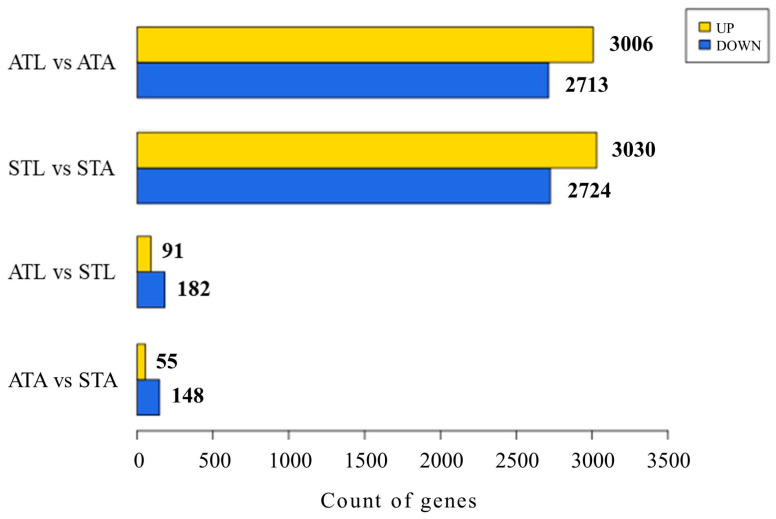
Differential gene expressions under different experimental conditions. The bar chart shows the count of differentially expressed genes (DEGs) categorized as upregulated (yellow) and downregulated (blue) for four different comparisons: ATL vs. ATAs, STL vs. STAs, ATL vs. STL, and ATAs vs. STAs. Differential expression analysis was conducted with criteria set at an absolute fold change (|FC|) of ≥1.5 and a statistical significance threshold of raw *p*-value < 0.05. ATL, acetone-treated honeybee larvae; ATAs, acetone-treated honeybee adults; STL, spinetoram-treated honeybee larvae; STAs, spinetoram-treated honeybee adults.

Using the criteria of fold change (FC) ≥ 1.5 and a raw *p*-value < 0.05, DEGs were identified in the following group comparisons: STL vs. STAs (5754 DEGs, 3030 upregulated and 2724 downregulated), ATL vs. ATAs (5719 DEGs, 3006 upregulated and 2713 downregulated), ATL vs. STL (273 DEGs, 91 upregulated and 182 downregulated), and ATAs vs. STAs (203 DEGs, 55 upregulated and 148 downregulated) (Figure 1). A complete list of DEGs is included in Supplementary Materials File S1. For all comparisons, heat maps were constructed using two-way hierarchical clustering with Z-scores for normalized values (log2-based) to group genes based on their expression profiles (Figure S1). This approach revealed the similarities and differences in gene expression patterns between samples. Figure S2 shows volcano plots for all comparisons. This visualization enabled the identification of the key genes that were significantly upregulated or downregulated in each comparison. Genes that were highly significant and showed significant changes in expression are positioned in the upper left (downregulated) or upper right (upregulated) corner of the graph.

### 2.2. Identification of Unique Genes Affected in Spinetoram-Exposed Honeybee Larvae and Adults

**Figure 2:**
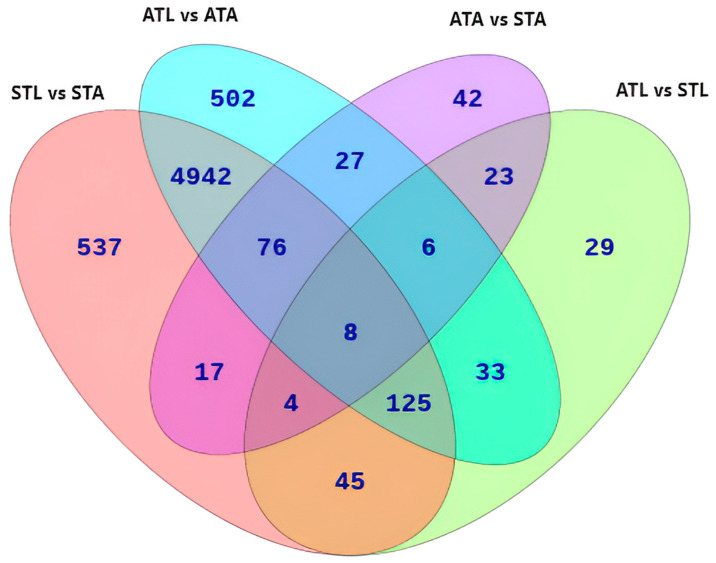
Venn diagram of DEGs across indicated comparisons. The diagram illustrates the unique and overlapping DEGs across the following comparisons, providing insights into gene expression changes under different treatments and developmental stages: STL vs. STAs, ATL vs. STL, ATL vs. ATAs, and ATAs vs. STAs. ATL, acetone-treated honeybee larvae; ATAs, acetone-treated honeybee adults; STL, spinetoram-treated honeybee larvae; STAs, spinetoram-treated honeybee adults.

A Venn diagram was constructed to understand the overlap or uniqueness of DEGs identified between the experimental groups (Figure 2). A total of 5151 DEGs overlapped between the ATL vs. ATA and STL vs. STA comparisons, suggesting that the expression of these genes is critical for the normal growth of honeybees. In addition to the 5151 common DEGs, 537 specific DEGs were identified for STL vs. STAs, and 502 specific DEGs were identified for ATL vs. ATAs. In response to SPI, 29 unique DEGs were identified in the larval stage, and 42 unique DEGs were identified in the adult stage. Furthermore, among unique DEGs, 23 DEGs were found to overlap between the ATL vs. STL and ATA vs. STA comparisons, potentially revealing critical genes that mediate the toxic effects of SPI exposure. The full list of genes that overlapped between groups and were specific to each group is provided in Supplementary Materials File S2.

### 2.3. GO Enrichment Analysis of Significant DEGs

Significant DEGs were used for GO enrichment analysis to categorize each comparison into three functional groups: biological processes (BPs), cellular components (CCs), and molecular functions (MFs).

#### 2.3.1. ATL vs. ATAs and STL vs. STAs

As most DEGs were common between ATL vs. ATAs and STL vs. STAs (Figure 2), GO terms related to BPs, CCs, and MFs overlapped between these comparisons (Figures S3 and S4).

CC terms primarily emphasized different aspects of mitochondrial architecture, underlining their critical roles in energy metabolism and signaling, which are essential for the normal development of honeybees. These common CC terms included mitochondrion, mitochondrial inner membrane, mitochondrial envelope, organelle inner membrane, organelle envelope, envelope, cytoplasm, and mitochondrial membrane. The uncommon CC categories identified included the proton-transporting two-sector ATPase complex, respirasome, mitochondrial protein-containing complex, and inner mitochondrial membrane protein complex, which suggested intricate cellular responses to SPI exposure. These categories reflect a specific cellular adaptation involving specialized compartments to mitigate mitochondrial dysfunction and cellular damage caused by SPI.

The MF terms that were commonly affected in both comparisons included transporter activity, transmembrane transporter activity, structural constituents of cuticle, passive transmembrane transporter activity, channel activity, active transmembrane transporter activity, monoatomic ion transmembrane transporter activity, and proton transmembrane transporter activity (Figures S3 and S4). These molecular functions, which include various types of transporter activities and structural roles, are crucial for maintaining cellular homeostasis and facilitating essential physiological processes, such as nutrient uptake, waste elimination, and protective barrier maintenance, thereby ensuring efficient cellular function and supporting organismal growth and survival. The results showed that Vitamin B6 binding and pyridoxal phosphate binding were specifically affected in the STL vs. STA comparison, suggesting that the bees activated biochemical changes essential for detoxification processes and neural function maintenance, ultimately supporting their resilience to SPI exposure. Vitamin B6 and its active form, pyridoxal phosphate, are coenzymes that are crucial for numerous enzymatic reactions, particularly amino acid metabolism, neurotransmitter synthesis, and gene expression modulation [38,39].

The common biological processes identified in both comparisons underscore fundamental cellular activities related to energy production and metabolism (Figures S3 and S4). These processes are essential for maintaining cellular homeostasis, supporting vital cellular functions, and ensuring the survival of honeybees, thereby facilitating their normal growth and development. Specifically, activities such as mitochondrial ATP synthesis, aerobic electron transport chain, the ATP biosynthetic process, proton motive force-driven ATP synthesis, and the ATP metabolic process were highlighted in both datasets, reflecting their critical roles in energy management within cells. BPs, such as small-molecule metabolic processes, monoatomic ion transmembrane transport, carboxylic acid metabolic processes, and oxoacid metabolic processes, were exclusively highlighted in the STL vs. STAs comparison, indicating a specific metabolic adaptation in honeybees to cope with SPI exposure. These adaptations are crucial for ensuring the survival and normal growth of bees by enhancing their capacity for detoxification and stress response to environmental challenges. The genes associated with each enriched GO term are listed in Tables S1 and S2 and ranked from the highest downregulation to the highest upregulation.

#### 2.3.2. ATL vs. STL

**Figure 3:**
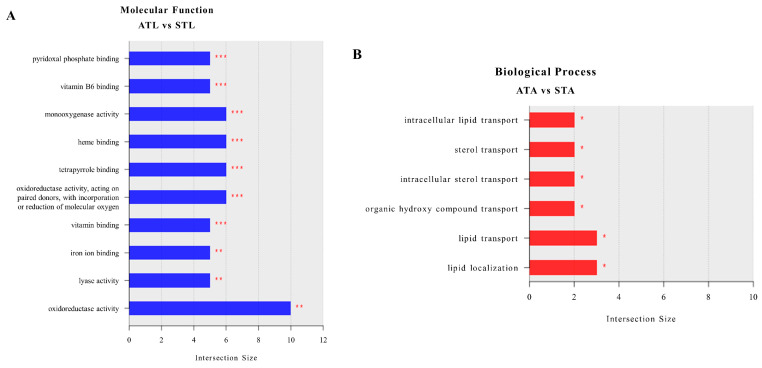
Top GO terms in different transcriptome comparisons. Top 10 molecular functions enriched in ATL vs. STL (**A**), and top 6 biological processes enriched in ATAs vs. STAs (**B**). ATL, acetone-treated honeybee larvae; ATAs, acetone-treated honeybee adults; STL, spinetoram-treated honeybee larvae; STAs, spinetoram-treated honeybee adults. * *p* < 0.05, ** *p* < 0.01, *** *p* < 0.001.

The MFs identified in the STL were significant, because they revealed critical disruptions in various metabolic and physiological processes (Figure 3A). The genes associated with each enriched GO term are listed in Table S3 and ranked from the highest downregulated gene to the highest upregulated gene. Altered pyridoxal phosphate and vitamin B6 binding suggest potential impairments in amino acid metabolism and enzyme function, which are essential for growth and development. Changes in monooxygenase and oxidoreductase activities indicate oxidative stress and modifications in detoxification processes, reflecting the larval response to SPI-induced oxidative damage. Alterations in heme and tetrapyrrole binding indicate disruptions in the respiratory processes and energy metabolism; whereas, changes in iron ion binding highlight the potential impacts on oxygen transport and DNA synthesis. The observed modifications in lyase activity further suggest metabolic pathway disturbances. Overall, these molecular changes indicate that pesticide exposure can lead to significant metabolic, enzymatic, and physiological stress in honeybee larvae, potentially affecting their development, health, and survival.

#### 2.3.3. ATAs vs. STAs

The results indicated that SPI exposure in adult honeybees significantly disrupted several critical biological processes related to lipid metabolism and transport (Figure 3B). The genes associated with each enriched GO term are listed in Table S4 and ranked from highest downregulation to highest upregulation. Specifically, GO terms were related to intracellular lipid transport, sterol transport, and intracellular sterol transport, which are essential for maintaining cell membrane integrity, energy balance, and hormone synthesis. Additionally, the transport of organic hydroxyl compounds, which are vital for metabolic processes and detoxification, was affected, potentially leading to the accumulation of toxic substances and metabolic imbalances. General lipid transport and localization impairment further highlight energy distribution and cellular function issues. These disruptions can compromise cellular stability, signaling pathways, and overall physiological health, severely affecting the ability of bees to perform essential functions and threatening their survival.

### 2.4. KEGG Pathway Enrichment Analysis of Significant DEGs

Based on the enrichment map test values, the enriched KEGG pathways were segregated into six major categories: metabolism, genetic information processing, environmental information processing, cellular processes, organismal systems, and human diseases. The complete list of all enriched biological pathways identified in the enrichment analysis is shown in Figure S5.

**Figure 4:**
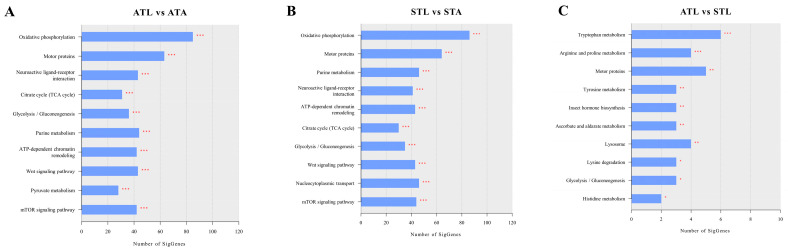
Top 10 KEGG pathways in different transcriptome comparisons. ATL vs. ATAs (**A**), STL vs. STAs (**B**), ATL vs. STL (**C**). ATL, acetone-treated honeybee larvae; ATAs, acetone-treated honeybee adults; STL, spinetoram-treated honeybee larvae; STAs, spinetoram-treated honeybee adults. * *p* < 0.05, ** *p* < 0.01, *** *p* < 0.001.

Among the top 10 enriched pathways in the ATL vs. ATA and STL vs. STA comparisons, nine pathways, including oxidative phosphorylation, motor proteins, glycolysis/gluconeogenesis, the neuroactive ligand–receptor interaction, the citrate cycle (TCA cycle), purine metabolism, ATP-dependent chromatin remodeling, the Wnt signaling pathway, and the mTOR signaling pathway, were enriched in both comparisons (Figure 4A,B). These results underscore the critical role that these pathways play in the normal development of honeybees, particularly in their metabolism, energy production, and cellular signaling. Furthermore, pyruvate metabolism was uniquely enriched in ATL vs. ATAs (Figure 4A); whereas, nucleocytoplasmic transport was distinct in STL vs. STAs (Figure 4B). The disruption of these pathways after SPI exposure suggests that SPI causes metabolic imbalances, energy deficits, and perturbations, potentially affecting honeybees’ normal survival. Tables S5 and S6 show the DEGs for each enriched KEGG pathway in ATL vs. ATAs and STL vs. STAs, respectively, ranked from the highest downregulation to the highest upregulation.

During the larval stage, exposure to SPI enriched several key biological pathways compared to the control group (Figure 4C). The top 10 enriched pathways were tryptophan metabolism, arginine and proline metabolism, motor proteins, tyrosine metabolism, insect hormone biosynthesis, ascorbate and aldarate metabolism, lysosomes, lysine degradation, glycolysis/gluconeogenesis, and histidine metabolism. These findings highlight the significant impact of SPI exposure on larval honeybees, particularly on energy production, stress responses, metabolism, hormonal regulation, and cellular repair. The genes associated with each enriched pathway in all comparisons are listed in Table S7 and ranked from the highest downregulation to the highest upregulation. A limited number of DEGs were identified in the adult stage following SPI exposure (Figure 1 and Figure 2); thus, no significant signaling pathways were identified through KEGG enrichment analysis.

### 2.5. Validation of Transcriptome Data Accuracy by qPCR

**Figure 5:**
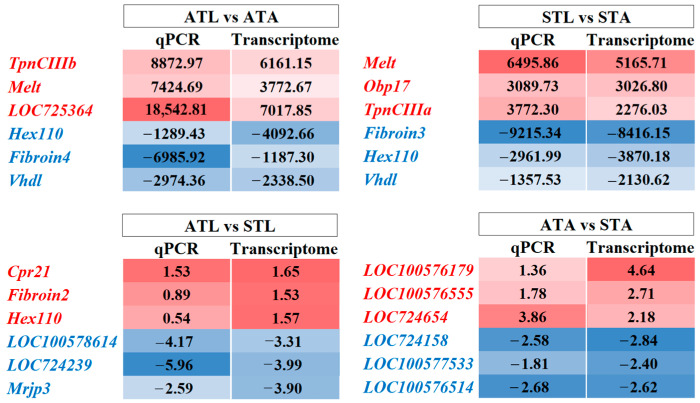
Comparison of gene expression data from qPCR and transcriptome analysis across various honeybee sample comparisons. The heatmaps display the fold change in expression levels of selected genes measured by qPCR, with the relative fold change obtained in transcriptome analysis shown adjacent. The red color indicates upregulated genes, and the blue color indicates downregulated genes. Comparisons include ATL vs. ATAs, STL vs. STAs, ATL vs. STL, and ATAs vs. STAs. ATL, acetone-treated honeybee larvae; ATAs, acetone-treated honeybee adults; STL, spinetoram-treated honeybee larvae; and STAs, spinetoram-treated honeybee adults.

To validate the transcriptome sequencing data, three up- and three downregulated genes were randomly selected from the top 30 DEGs in each comparison, and qPCR was conducted. The qPCR analysis of the tested DEGs revealed an expression pattern that aligned with the RNA sequencing data (Figure 5), confirming the reliability of the transcriptome dataset. Both upregulated and downregulated genes in the RNA-seq data maintained their respective expression trends in the qPCR validation, reinforcing the robustness of the transcriptomic findings.

## 3. Discussion

The global decline in honeybee populations underscores the necessity to investigate how pesticides disrupt ecosystems [29,40]. Honeybees forage over kilometers for nectar and pollen vital to their colony [41], but pesticide use contaminates these resources, harming the hive and exposing bees to pesticides. Pesticides interfere with various biological processes in target and nontarget organisms beyond their intended effects [34,36,37]. Such exposure disrupts biological processes, leading to delayed development, reduced adult lifespan, premature shifts in hive roles, and disrupted foraging activities [8,28,42].

Our recent study showed that exposure to SPI can lead to severe developmental deformities and alterations in detoxification and behavioral enzymes in honeybees [28]. These findings emphasize the need for comprehensive molecular-level investigations using advanced technologies, such as next-generation transcriptome sequencing, which provides a detailed molecular overview beyond the limitations of traditional methods [33,34,36,37]. We revealed significant alterations in gene expression by conducting whole-transcriptome sequencing of honeybees at both the larval and adult stages after chronic sublethal SPI exposure during the larval phase. Specifically, 29 and 42 genes were uniquely affected in larvae and adults, respectively, with 23 genes overlapping between the larval and adult comparisons. This overlap suggests key genes that may mediate SPI toxicity across both developmental stages, potentially disrupting critical molecular pathways essential for honeybee development and survival. GO and KEGG pathway analyses of the DEGs indicated that SPI targets the critical honeybee pathways essential for development and behavior. The identification of stage-specific molecular targets of SPI toxicity underscores the urgent need for further research to thoroughly comprehend their impacts on honeybee health and colony dynamics.

### 3.1. SPI-Induced Perturbations in Gene Expression and Biological Pathways in Honeybee Larvae

The findings from this study revealed that SPI affects honeybee larval metabolism by disrupting several crucial signaling pathways (Figure 4), likely contributing to the deformities observed in our previous study [28]. For example, arginine and proline metabolism compounds can affect detoxification, immune defense, stress tolerance, cellular repair, and overall metabolic homeostasis [43,44]. Changes in tyrosine metabolism can lead to abnormalities in energy metabolism, neurotransmitter and protein synthesis, and cellular repair [45]. Disruptions in ascorbate and aldarate metabolism may compromise the antioxidant defense and efficient detoxification processes [46]. For example, the upregulation of the putative aldehyde dehydrogenase family 7 member A1 homolog in this pathway likely represents an adaptive phase I detoxifying response to reduce the immediate risks associated with SPI exposure [47]. The unexpected downregulation of UDP-glucuronosyltransferase after SPI exposure, despite its crucial role in phase II detoxification, particularly in response to pesticides, highlights the susceptibility of larvae to SPI [48,49]. The perturbation of histidine metabolism disrupts the immune and inflammatory responses, pH, metal ion balance, protein synthesis, enzyme activity, and antioxidant defenses [50]. The disruption of lysine degradation affects energy production, detoxification, and immune functions [51]. The widespread disruption of essential metabolic pathways highlights the profound negative effects of SPI on larval health and suggests that SPI exposure could significantly affect larval development, immune competence, and overall viability. These adverse effects highlight the need for strict regulation of SPI use and the development of safer alternatives to protect honeybee populations and ensure their sustainability.

Tryptophan metabolism was identified as the metabolic pathway most affected by SPI treatment. This dysregulation has also been observed at sublethal doses of acetamiprid [52], negatively affecting the honeybee larvae’s development, emergence rate, and lifespan [53]. Zhang et al. emphasized the crucial role of tryptophan metabolism in improving memory behavior [54], and its supplementation regulates growth, development, and various physiological and biochemical properties in worker bees [55], underscoring its significance in larval development and overall health. Tryptophan 2,3-dioxygenase (TDO) and kynurenine/alpha-aminoadipate aminotransferase, mitochondrial (KAT) were downregulated in this pathway. TDO catalyzes the first rate-limiting step in the kynurenine pathway, the major route of tryptophan catabolism, and leads to the conversion of tryptophan into N-formylkynurenine [56]. KAT catalyzes the transamination of kynurenine and alpha-aminoadipate, which are intermediates in the tryptophan and lysine catabolic pathways, respectively [51]. The downregulation of TDO and KAT likely leads to tryptophan accumulation, the disruption of the metabolic balance, and the increased production of serotonin, dopamine, norepinephrine, and epinephrine. This is evidenced by the upregulation of aromatic-L-amino-acid decarboxylase (AADC), a crucial enzyme in the production of these neurotransmitters [57,58] in STL. Serotonin is associated with the regulation of foraging behavior, with higher levels often observed in foragers than in hive-bound bees [59]. Dopamine and serotonin are essential for learning and memory in honeybees [58,60]. Overall, the perturbations in tryptophan metabolism in honeybee larvae may disrupt neurotransmitter synthesis, gene regulation, and immune responses, thereby affecting the colony’s health and dynamics.

Another significant finding in SPI-exposed larvae was the perturbation of the insect hormone biosynthesis pathway, which can lead to developmental abnormalities, reproductive issues, and altered behavior in honeybees and can affect colony dynamics [61,62,63]. In our study, we observed the upregulation of genes such as juvenile hormone acid methyltransferase (JHAMT), retinol dehydrogenase 1 (RALDH1), and methyl farnesoate epoxidase. The juvenile hormone (JH) pathway is crucial in the development, caste determination, reproduction, and behavior of honeybees [62,63]. As the rate-limiting enzyme that catalyzes the final step of JH biosynthesis, JHAMT is pivotal in all these regulatory pathways [64,65]. RALDH1 is essential for retinoid metabolism and is involved in the conversion of retinol to retinaldehyde, an important step in retinoic acid synthesis [66]. The upregulation of RDLH1, which is critical for the phototransduction cascade [66], can profoundly affect bee behavior, navigation, foraging, and social interaction within the hive, owing to its role in visual perception. Considering its pivotal role in regulating gene expression [67], the upregulation of RDLH1 may also affect cell proliferation, differentiation, and immune responses at the cellular level in honeybees. Overall, exposure to SPI during the larval stage of honeybees disrupts hormone biosynthesis pathways, particularly affecting JH and retinoid metabolism, which are critical for development, behavior, and survival. The upregulation of these key enzymes could lead to developmental defects and behavioral changes, ultimately affecting colony health and dynamics.

In STL, various motor proteins were downregulated, including dynein intermediate chain 3 (ciliary), dynein heavy chain 7 (axonemal-like), dynein heavy chain 6 (axonemal), dynein heavy chain 7 (axonemal), and kinesin 2C. Kinesins and dyneins are pivotal for intracellular transport, facilitating essential cellular functions, such as neurotransmitter transport, protein trafficking, and organelle positioning [68,69]. Kinesins serve as molecular motors that facilitate the directional transport of diverse cargo, such as membranous organelles, protein complexes, and mRNAs [69]. Conversely, dyneins transport materials toward the microtubule minus ends, moving them from the cell periphery to the cell interior [68]. Overall, the observed downregulation of these motor proteins in STL highlights the potential of SPI to disrupt essential cellular functions, which could significantly affect cellular health and organism development. Further research is needed to elucidate the precise mechanisms by which SPI influences motor protein expression and to examine the broader implications of these findings for the physiology and development of organisms.

Another pathway enriched in STL was the lysosome pathway, which is crucial for maintaining cellular homeostasis in honeybees by eliminating damaged organelles and proteins, thereby promoting cell health and longevity [70]. SPI exposure increased the expression of four proteins, namely Lipase3, a putative inorganic phosphate cotransporter (PiC), a putative glucosylceramidase (GCase), and arylsulfatase B, transcript variant X3 (ARSB-X3). Lipases, such as Lip3, break down triacylglycerol (TAG) into fatty acids (FAs) for energy production and membrane lipid synthesis during energetic stress [71,72]. Therefore, Lip3 overexpression can enhance TAG breakdown and increase the energy and resources required for membrane lipid synthesis. Similarly, an increase in PiC expression can disturb phosphate balance, affecting energy metabolism and overall cellular function [73]. GCase is crucial for degrading glucosylceramide into glucose and ceramide and maintaining membrane structure and signaling [74]. Increased GCase levels may enhance this degradation, supporting membrane integrity and proper signaling pathways [74]. Furthermore, ARSB, a lysosomal enzyme, hydrolyzes sulfate ester bonds in glycosaminoglycans, steroids, and xenobiotics, maintaining metabolic balance and aiding in the elimination of sulfated metabolites [74]. The overexpression of ARSB increases hydrolysis, enhances the elimination of sulfated metabolites, and supports detoxification and metabolic balance [74]. Overall, although the overexpression of these lysosomal proteins can provide some advantages in terms of stress resistance and metabolic efficiency, it could also pose challenges related to metabolic balance and resource allocation. Therefore, further investigation of these aspects is necessary to fully understand the effects of SPI exposure on honeybee larval health.

Martelli et al. (2022) demonstrated that low doses of spinosad antagonize nAChRs, leading to lysosomal storage disorders, mitochondrial dysfunction, oxidative stress, and impaired lipid metabolism [15]. Similarly, Martelli et al. (2023) identified transcriptomic signatures linked to oxidative stress, immune function, energy metabolism, and lysosomal activity [16]. Given the similarities in pesticide classes, SPI may cause comparable dysfunctions in honeybees. This is supported by the enrichment of the lysosome pathway, particularly the upregulation of Lipase3 (involved in lipid metabolism) [71,72], PiC (critical for ATP synthesis) [73], GCase (for glucocerebroside breakdown) [74], and ARSB-X3 (in lysosomal glycosaminoglycan degradation) [74], which are indicators of lysosomal disruption in SPI-exposed honey bees. Additionally, the enrichment of arginine and proline metabolism, tryptophan metabolism, and ascorbate and aldarate metabolism in the transcriptome of SPI-exposed honey bees indicates a heightened oxidative stress response and disruptions in cellular homeostasis [75]. These pathways are critical for maintaining mitochondrial integrity and managing oxidative stress. Therefore, it is plausible that the disruption of these pathways in response to SPI may suggest that SPI exposure leads to the disruption of lysosomal and mitochondrial functions in honeybees.

The deformed wings (DWs) and deformed antennae (DA) observed in SPI-exposed honey bees [28] can be attributed to disruptions in several key biological pathways observed in its transcriptome. Most notably, the insect hormone biosynthesis pathway, crucial for regulating metamorphosis and the differentiation of imaginal disks into wings and antennae [76,77], was affected by SPI. Normally, JH maintains the larval state, and its reduction is needed for proper development during the pupal stage [76,77]. Elevated JH levels in SPI-exposed bees may prevent this, leading to DWs and DAs. The arginine and proline metabolism pathway, important for collagen formation and cuticle strength [43,78], was also impacted by SPI. This may weaken the cuticle and wing membranes, causing malformations. Additionally, the tryptophan metabolism pathway, which governs neurotransmitter production and neural patterning, particularly in antenna development [76,79], was also disrupted, leading to defective sensory organs. Other pathways, such as those involved in intracellular transport, pigmentation, waste degradation, and protein synthesis, may also contribute to these deformities. Understanding the interactions between these pathways is key to explaining the phenotypic abnormalities seen in SPI-exposed bees.

### 3.2. Responsive Genes in Adult Honeybees Following Spinetoram Exposure

Our study revealed that SPI-exposed honeybees showed upregulation in several crucial metabolic and sensory proteins during the adult phase. Notably, glucose dehydrogenase [FAD, quinone] (GDH), which plays a vital role in cellular energy metabolism by catalyzing the oxidation of glucose to gluconolactone and its subsequent hydrolysis to gluconic acid [80], showed increased expression. This aligns with previous studies indicating that exposure to pesticides, such as cypermethrin, clothianidin, imidacloprid, and thiamethoxam, similarly upregulates GDH in honeybees [81,82], suggesting that it is a part of their response to xenobiotic stress. Additionally, the increased expression of the glutamate receptor 1-like transcript variant X2 reveals potential disruptions in honeybee neuronal function and behavior [83]. The upregulation of odorant receptor 30a, transcript variant X1, suggests alterations in olfactory perception, foraging behavior, social communication, and environmental adaptation [84]. The observed upregulation of cytochrome b561 domain-containing protein 2, cytochrome b5, probable cytochrome P450 304a1, glutamate receptor 1-like, transcript variant X2, and larval-specific VHDL indicated a multifaceted molecular response to SPI exposure involving detoxification and metabolic adaptation [85]. These findings underscore the significant role of metabolic and sensory protein upregulation in the adaptive responses of honeybees to environmental stressors, particularly pesticide exposure.

This study revealed that adult honeybees exposed to SPI undergo significant downregulation in several critical proteins that are essential for cellular and colony health. Notably, X-ray repair cross-complementing protein 4, which is essential for DNA repair [86], and MAD2B (mitotic spindle assembly checkpoint protein), which is crucial for chromosome stability during cell division [87], were downregulated. This downregulation may increase genomic instability, resulting in increased disease susceptibility, reduced reproductive capacity, and accelerated colony aging. Reduction in the ubiquitin-protein ligase TRAIP, which is involved in protein degradation, DNA repair, and the cellular stress response [88], could adversely affect colony longevity and health. Additionally, a reduction in RNA-binding proteins disrupts RNA metabolism [89], thereby affecting the stress response and immune function. Furthermore, the downregulation of FAST kinase domain-containing protein 4, which is integral to mitochondrial RNA processing and apoptosis [90], possibly leads to mitochondrial dysfunction and increased cell death. The downregulation of oligoribonucleases, which degrade short RNA oligomers [91], increases the accumulation of RNA fragments, further compromising bee health. Another crucial downregulated gene in SPI-exposed adult honeybees is Myb-like protein D, a transcription factor that regulates the expression of genes necessary for cell cycle progression, differentiation, and stress response [92]. This downregulation may result in disrupted cell cycle progression, impaired differentiation, and a diminished stress response. The downregulation of transmembrane protein 70 homolog hampers ATP synthase complex assembly, impairing ATP production critical for energy metabolism [93]. Such downregulation likely signals mitochondrial dysfunction, which affects flight endurance, activity levels, and hive temperature regulation, possibly contributing to CCD and population declines [6]. The downregulation of ADP-ribosylation factor-like protein 2-binding protein-like disrupts small GTPase regulation [94], eventually affecting cellular activities, stress responses, and behavioral patterns in honeybees. The cumulative effect of the downregulation of key proteins involved in DNA repair, stress response, and energy metabolism likely leads to increased disease susceptibility, impaired reproductive performance, and accelerated aging within the colony. Consequently, these factors contribute to a reduction in the bee population.

### 3.3. Critical Genes and Pathways Essential for Honeybee Normal Growth and Development

The 5151 DEGs common to the ATL vs. ATA and STL vs. STA comparisons may play key roles in the normal development of honeybees (Figure 2). Therefore, a significant overlap was observed among the ten most enriched pathways in the ATL vs. ATA and STL vs. STA comparisons, indicating that these pathways play critical roles during the normal growth and in stage-specific functions. The transition from larvae to adults requires a higher energy supply owing to physiological changes, including tissue growth, differentiation, and flight muscle development [95]. The enrichment of the TCA cycle, a fundamental metabolic pathway that accounts for most of the cellular energy supply, and the oxidative phosphorylation pathway, which efficiently produces ATP, provides the necessary energy to support these processes and ensure the successful growth from larvae to adults in honeybees [96]. Furthermore, the commonly enriched Wnt and mTOR signaling pathways regulate cell growth, proliferation, survival, and metabolism [97,98,99]. The enrichment of the ATP-dependent chromatin remodeling pathway highlights the crucial role of regulating gene expression, DNA repair, and other chromatin-related processes in honeybee growth and development [100]. Additionally, the enrichment of motor proteins indicates the key role of intracellular transport, which is essential for the physiological processes required for normal growth and maintenance of overall cell homeostasis [68,69]. The enrichment of purine metabolism suggests that increased DNA and RNA synthesis is necessary for normal growth; whereas, the enrichment of neuroactive ligand–receptor interactions indicates the significance of neural signaling in developmental processes and behavior [101,102]. The disruption of the glycolysis/gluconeogenesis pathway suggests the role of energy production in the growth of honeybees. The enrichment of similar KEGG pathways does not necessarily imply the same function in these comparisons. A single gene change within any pathway may lead to opposite outcomes. Therefore, detailed comparative studies of these common pathways can provide insights into the specific gene functions and interactions that are critical for honeybee development under different conditions.

Nucleocytoplasmic transport was specifically disrupted in the STL compared to that in the STAs, highlighting its alteration due to SPI exposure in honeybees. The downregulation of 54 genes associated with nucleocytoplasmic transport indicated a severe alteration of this pathway in the STA group. This may affect mRNA export for protein synthesis, protein import, and the regulation of gene expression [103]. The downregulation of a subset of genes and the 14 upregulated genes might also represent a strategic adjustment to limit the influx of harmful molecules and the efflux of essential molecules, thereby protecting against SPI toxicity. Based on these DEGs, we can assume that SPI exposure alters nuclear transport in a manner distinct from that observed in normal cells, leading to disruptions in cellular homeostasis and consequently impacting the overall health of honeybees. Further research is required to elucidate the specific roles of the upregulated and downregulated genes in the nucleocytoplasmic transport pathway.

This study used chronic sublethal exposure to SPI to model its effects on honeybee health. However, in real-world environments, bees are simultaneously exposed to pesticides, pathogens, phytochemicals, and nutrient deficiencies that can amplify or alter their effects on bee health. For example, Ardalani et al. revealed that dietary phytochemicals reduced pesticide residues in honeybees [104]. However, the controlled experimental design used in this study provides a crucial foundation for elucidating the specific mechanisms of SPI toxicity and guiding future studies in more complex environmental contexts. The study results revealed the molecular mechanisms of SPI toxicity at the transcriptome level; however, proteomic analysis, phenotype assessment, and more authentic environmental conditions are needed to fully understand its effects on bee health and colony dynamics. Despite these limitations, this study established a solid foundation for further investigation and identified the key molecular pathways to be considered in pesticide screening to mitigate their detrimental effects on honeybees.

## 4. Materials and Methods

### 4.1. Honeybee In Vivo Rearing and Spinetoram Exposure

First-instar honeybee (*Apis mellifera* L.) larvae were harvested from healthy colonies situated in an experimental apiary within the National Institute of Agricultural Sciences, Rural Development Administration, Republic of Korea (35.591° N, 126.278° E). Strict precautions were taken one month before beginning the experiments to ensure that the bees were not contaminated with other chemicals or pesticides. Age-matched larvae were obtained by introducing the queen into empty combs for oviposition in the hive three days before grafting. The experimental procedures were based on previous studies [8,28]. First-instar larvae were grafted into 48-well plates (SPL, Pocheon-si, Gyeonggi-do, Republic of Korea) using a grafting tool and fed with 20 µL of diet A. No food was provided the following day. On day 3, each larva was provided with 20 µL of diet B. On days 4, 5, and 6, the larvae were fed 30, 40, and 50 µL of diet C, respectively. The specific compositions of diets A, B, and C are listed in Table 2. During the experiments, except during the feeding period, bees were maintained in darkness at a constant temperature of 35 °C (STH E-800; Dahin Scientific, Daegu-si, Gyeongsang-do, Republic of Korea). The larvae were maintained in desiccators (5317-0180, Nalgene, Rochester, NY, USA) to regulate the relative humidity (RH), which was adjusted to 95 ± 5% with saturated potassium sulfate from days 1 to 8. The RH was set to 80 ± 5% during the pupal stage using saturated sodium chloride from days 8 to 15. On day 15, the pupae were transferred to emergence boxes containing a 50% (*w*/*v*) sugar solution and maintained at 60% RH.

Honeybee larvae were exposed to SPI following the protocols outlined in our previous studies [8,28]. The selected dosage for SPI was 0.0017 µg a.i./larva, which is one-tenth of the LD_50_ value determined in our study [28]. SPI (DRE-C16972770, 88.84% purity; Dr. Ehrenstorfer, Wesel, North Rhine-Westphalia, Germany) stocks were prepared in acetone and dissolved in the diet to achieve the required concentrations. During the exposure period from day 3 to 6 post-grafting, each larva received a total of 0.0017 µg of SPI. The acetone concentration was maintained at 0.5% in the SPI exposure group, and larvae exposed to a diet containing 0.5% acetone were used as the control group. The deceased larvae were removed daily. Healthy larvae and adults were collected on days 8 and 19, respectively. Three replicate samples were collected for transcriptomic analysis and qPCR validation for each experimental condition. Each sample comprised three individual larvae or adults. All samples were rapidly frozen in liquid nitrogen and then stored at −80 °C for later analysis.

### 4.2. Sampling, cDNA Library Construction, DEG Selection, and Statistical Analysis

RNA extraction, quantification, integrity assessment, and cDNA library preparation were carried out following established protocols described in previous studies [36,37]. Briefly, total RNA extraction was performed using the QIAzol^®^ Lysis Reagent (#79306, QIAGEN, Hilden, Germany) followed by the RNeasy^®^ Mini Kit (#74106, QIAGEN, Hilden, Germany). Total RNA concentration was measured using Quant-IT RiboGreen (#R11490, Invitrogen, Waltham, MA, USA), and RNA integrity was evaluated with the TapeStation RNA Screentape (#5067-5576, Agilent, Santa Clara, CA, USA). RNA libraries were constructed using only high-quality RNA preparations with RIN values exceeding 7.0. The library was prepared using 1 μg of total RNA from each sample with the Illumina TruSeq Stranded mRNA Sample Prep Kit (Illumina, #RS-122-2101, San Diego, CA, USA). mRNA enrichment with poly-A tails was achieved using poly-T-attached magnetic beads. mRNA was fragmented using divalent cations at elevated temperatures and reverse-transcribed into first-strand cDNA using SuperScript II reverse transcriptase (#18064014; Invitrogen, Carlsbad, CA, USA) and random primers. The second-strand cDNA was synthesized using DNA polymerase I, RNase H, and dUTP. The cDNA fragments were subjected to end repair, A-tailing, and adapter ligation. The prepared products were purified and amplified by PCR to obtain a cDNA library. The completed libraries were quantified using KAPA Library Quantification kits (#KK4854, KAPA Biosystems, Wilmington, MA, USA), according to the qPCR Quantification Protocol Guide and quality-checked on the TapeStation system with a D1000 ScreenTape (#5067-5582, Agilent Technologies, Santa Clara, CA, USA).

The indexed cDNA libraries were sequenced using the NovaSeq platform (Illumina, Inc., San Diego, CA, USA) in a paired-end configuration (2 × 100 bp). To refine the sequence data, adapter sequences were removed, and low-quality bases were trimmed using Trimmomatic v0.38 [105]. The processed reads were aligned to the *Apis mellifera* genome (Amel_HAv3.1) using HISAT v2.1.0, based on HISAT and Bowtie2 implementations [106]. The resulting data in SAM file format were organized and indexed using SAMtools v1.9. Subsequent transcript assembly and quantification were performed using StringTie v2.1.3b [107]. Quantification at both the gene and transcript levels was performed by measuring raw read counts, fragments per kilobase of transcript per million mapped reads (FPKM), and transcripts per million (TPM).

### 4.3. DEG Selection

DESeq2 v1.24.0 [108] was employed for the statistical analyses of differential gene expression using raw counts as input data. During the quality control step, genes with nonzero counts in all replicates within at least one group were retained. Principal component analysis (PCA) and multidimensional scaling (MDS) plots were generated to verify the consistency of expression patterns across samples. The dataset was normalized using relative log expression (RLE) to account for variations in library size among samples. Differential gene expression was evaluated using the negative binomial Wald test provided by DESeq2 [108], from which fold changes and *p*-values were derived. *p*-values were adjusted using the Benjamini–Hochberg procedure to control for false discovery rate (FDR). The significant gene list was filtered based on |fold change| ≥ 1.5 and raw *p*-value < 0.05 criteria. The hierarchical clustering of significant genes was conducted on log-transformed data using Euclidean distance as the metric and complete linkage as the method.

### 4.4. DEG Analysis

Gene-enrichment and functional annotation analyses of significant genes were conducted using gProfiler (https://biit.cs.ut.ee/gprofiler/orth, accessed on 23 January 2024) [109] against the gene ontology (GO) and KEGG pathway databases. Adjusted *p*-values were obtained from the gProfiler results using a one-sided hypergeometric test and corrected using the Benjamini–Hochberg method [110].

### 4.5. Quantitative Reverse Transcription-Polymerase Chain-Reaction-Based Validation of Transcriptome Data

RNA extraction, cDNA preparation, and qPCR were performed, as described in our previous studies [34,37]. RNA extraction was performed in triplicates for each test condition. The larvae or adults were homogenized using the kimble^®^ pellet pestle^®^ cordless motor (DWK Life Sciences, Millville, NJ, USA) with a compatible pestle (SL.Tub3101.1, Scilab, Daejon, Chungcheongnam-do, Republic of Korea). An amount of 50 mg of larval or adult homogenate were carefully transferred to a 1.5 mL tube (Eppendorf, Hamburg, Germany) and lysed by adding 500 µL of Trizol-plus solution (Progen Life Sciences, New York, NY, USA; #PG1117). The solution was centrifuged at 8000*× g* for 3 min at 4 °C. The supernatant was transferred to a designated column in the Direct-zol RNA Miniprep Kit (R2052, Zymo Research, Irvine, CA, USA). RNA extraction was performed according to the manufacturer’s instructions. Notably, the kit included a DNase treatment step; therefore, no additional DNase treatment was applied. Quality assessment of the RNA was performed using Nanodrop 2000 (Thermo Fisher Scientific, Waltham, MA, USA). RNA (1500 ng/reaction) was converted into cDNA using AccuPower^®^ RocketScript™ Cycle RT PreMix (K-2205, Bioneer, Daejeon, Chungcheongnam-do, Republic of Korea).

Quantitative PCR (qPCR) analysis was performed using the AccuPower^®^ 2X GreenStar™ qPCR Master Mix (K6251, Bioneer, Daejeon, Chungcheongnam-do, Republic of Korea) on the CFX96 Dx real-time PCR detection device (Bio-Rad, Hercules, CA, USA). The qPCR was performed according to the manufacturer’s protocol. The cDNA concentration was consistently 30 ng in a 20 µL final PCR reaction across all tested samples. The concentration of each oligo primer was maintained at 10 picomoles per reaction, and the annealing temperature was set to the average of the forward and reverse primer melting temperatures (Table S8). No-template controls were included in triplicate for each oligo primer set.

The qPCR was conducted under the following conditions: initial denaturation at 95 °C for 5 min, followed by 45 cycles of denaturation at 95 °C for 10 s, annealing at the specified temperature (based on primer melting temperatures, Table S8) for 10 s, and extension at 60 °C for 10 s. Melting curve analysis was performed from 65 °C to 95 °C in 0.5 °C increments to confirm the specificity of the PCR products. The Ct values were obtained using three biological replicates for each test condition, and three technical replicates were performed for each sample. Gene expression fold changes were determined using the 2^−ΔΔCT^ method [111], with RPL13a as the normalization housekeeping gene. Detailed information on the oligonucleotide primers used for qPCR is provided in Table S8.

## 5. Conclusions

Our study revealed significant transcriptomic changes in honeybees across different developmental stages following SPI exposure. We identified DEGs linked to key biological processes, including lipid transport, hormone biosynthesis, and oxidative phosphorylation, underscoring the intricate effects of SPI on honeybee physiology. These disruptions in energy production, increased apoptosis, and impaired stress responses collectively weaken colonies, reduce productivity and resilience, and increase vulnerability to environmental stresses, diseases, and CCD. By identifying the specific signaling pathways disrupted by SPI, this study enhances our understanding of the impact of pesticides on key pollinators and provides a foundation for developing strategies to enhance honeybee resilience. Protecting honeybee populations and their ecosystems relies on integrating these molecular insights into comprehensive conservation practices that are crucial for maintaining crop production and biodiversity. By elucidating these pathways, we can better safeguard honeybee health and sustainability, ensuring their vital role as essential pollinators and maintaining ecological balance.

## Figures and Tables

**Table 1:** Transcriptome data summary showing the number of reads, sequence quality, and mapping rate.

Samples	Total	Clean	GC	Q20	Q30	Mapped	Mapped Rate	Unmapped Rate
	Reads	Reads	(%)	(%)	(%)	Reads	(%)	(%)
ATL-1	62,347,274	60,872,770	37.62	98.77	97.7	59,142,506	97.16	2.84
ATL-2	50,206,390	48,908,502	38.75	98.53	97.73	47,621,910	97.37	2.63
ATL-3	52,455,156	50,888,552	38.27	98.5	97.67	49,363,367	97.00	3.00
ATA-1	62,326,854	60,905,516	37.71	98.75	97.73	59,163,527	97.14	2.86
ATA-2	57,545,836	56,071,312	37.18	98.72	97.48	54,287,287	96.82	3.18
ATA-3	55,289,460	54,121,612	37.86	98.83	97.78	52,478,090	96.96	3.04
STL-1	62,875,890	61,453,462	38.55	98.79	97.75	59,736,568	97.21	2.79
STL-2	59,566,476	58,262,340	38.03	98.81	97.78	56,693,459	97.31	2.69
STL-3	57,889,536	56,640,604	38.93	98.81	97.85	55,043,771	97.18	2.82
STA-1	58,559,794	57,447,438	37.26	98.93	97.84	55,720,174	96.99	3.01
STA-2	49,519,968	48,424,000	38.32	98.62	97.83	47,142,930	97.35	2.65
STA-3	63,951,928	62,143,024	38.26	98.5	97.68	60,623,289	97.55	2.45

ATL, acetone-treated honeybee larvae; ATA, acetone-treated honeybee adult; STL, spinetoram-treated honeybee larvae; STA, spinetoram-treated honeybee adult; GC, guanine and cytosine; Q20, quality score 20; Q30, quality score 30.

**Table 2:** Composition of diets A, B, and C for honeybee larvae feeding.

Diet Name	Composition
Diet A	50% royal jelly, 6% D-glucose, 6% D-fructose, 37% distilled water, and 1% yeast extract
Diet B	50% royal jelly, 7.5% D-glucose, 7.5% D-fructose, 33.5% distilled water, and 1.5% yeast extract
Diet C	50% royal jelly, 9% D-glucose, 9% D-fructose, 30% distilled water, and 2.0% yeast extract

## Data Availability

All the data supporting the findings of this study are available from the corresponding author upon reasonable request.

## Supplementary Materials

The following supporting information can be downloaded at: https://www.mdpi.com/article/10.3390/ijms252211923/s1.
