# Supplementary material for: Molecular Impact of Sublethal Spinetoram Exposure on Honeybee (Apis mellifera) Larval and Adult Transcriptomes
Source: Int J Mol Sci. 2024 Nov 6;25(22):11923. doi: 10.3390/ijms252211923 (PMC11593601; doi:10.3390/ijms252211923)
Supplement: Supplementary file 1 [file ijms-25-11923-s001.zip › Supplementary FIle.pdf]

Supplementary data

Molecular impact of sublethal spinetoram exposure on honeybee (*Apis mellifera*) larval and adult transcriptomes

Bala Murali Krishna Vasamsetti, Juyeong Kim, Kyongmi Chon*, Bo-Seon Kim, Chang-Young Yoon, Sojeong Hwang, and Kyeong-Hun Park.

Toxicity and Risk Assessment Division, Department of Agro-Food Safety and Crop Protection, National Institute of Agricultural Sciences, Rural Development Administration, Iseo-myeon, Wanju-gun 55365, Jeollabuk-do, Republic of Korea

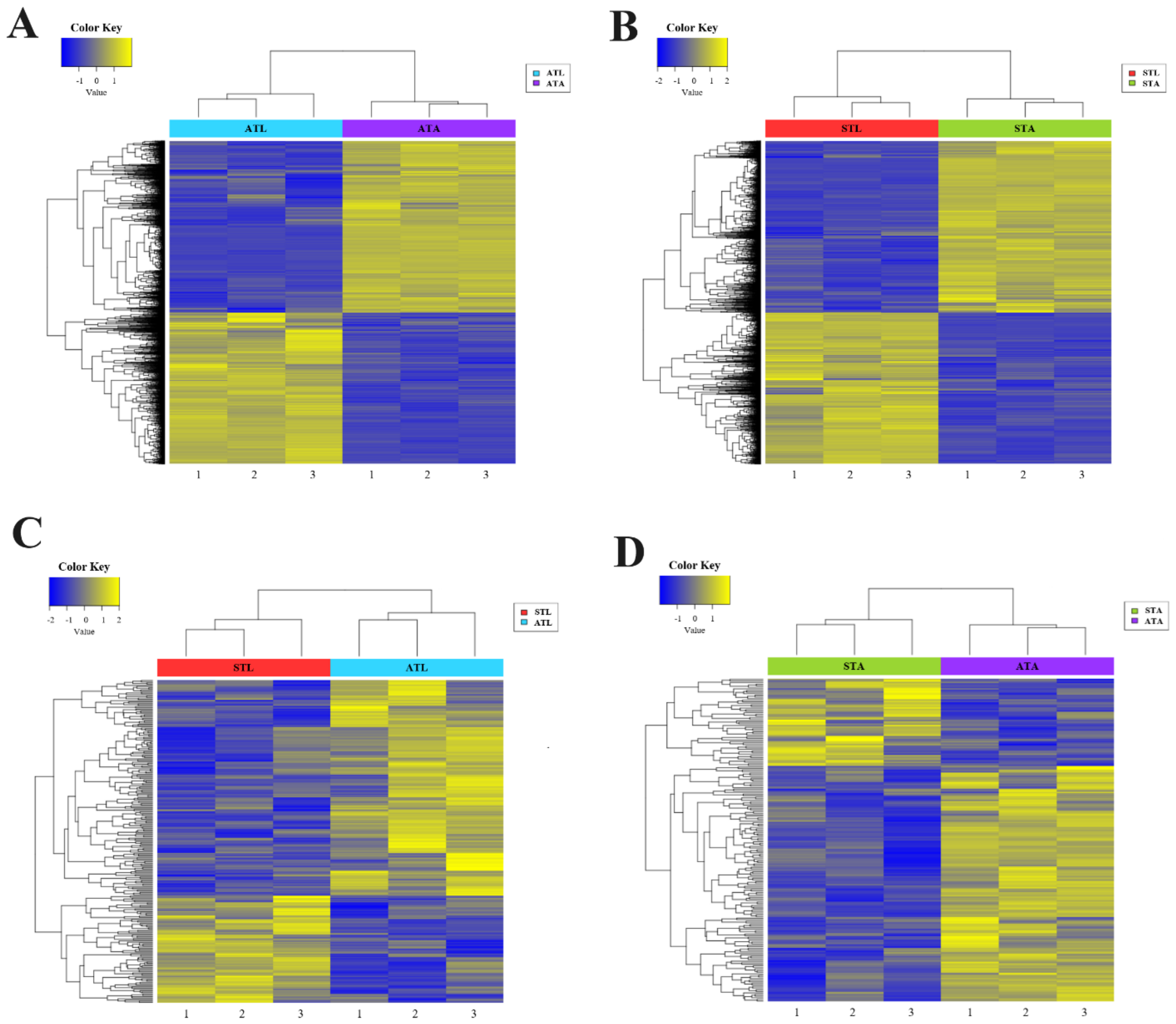

Figure S1: Comparative transcriptomic analyses of honeybee responses to spinetoram in honeybee larvae and adults. Heat map illustrates the differential expression of genes between four treatment comparisons: (A) ATL vs ATA, (B) STL vs STA, (C) ATL vs STL, (D) ATA vs STA. The data are presented as z-scores of normalized log2-transformed expression values. Two-way hierarchical clustering was applied to organize the data. Gene expression differences are considered significant at a fold change of 1.5 and with significance threshold p-values applied. ATL, acetone-treated honeybee larvae; ATA, acetone-treated honeybee adults; STL, spinetoram-treated honeybee larvae; STA, spinetoram-treated honeybee adults.

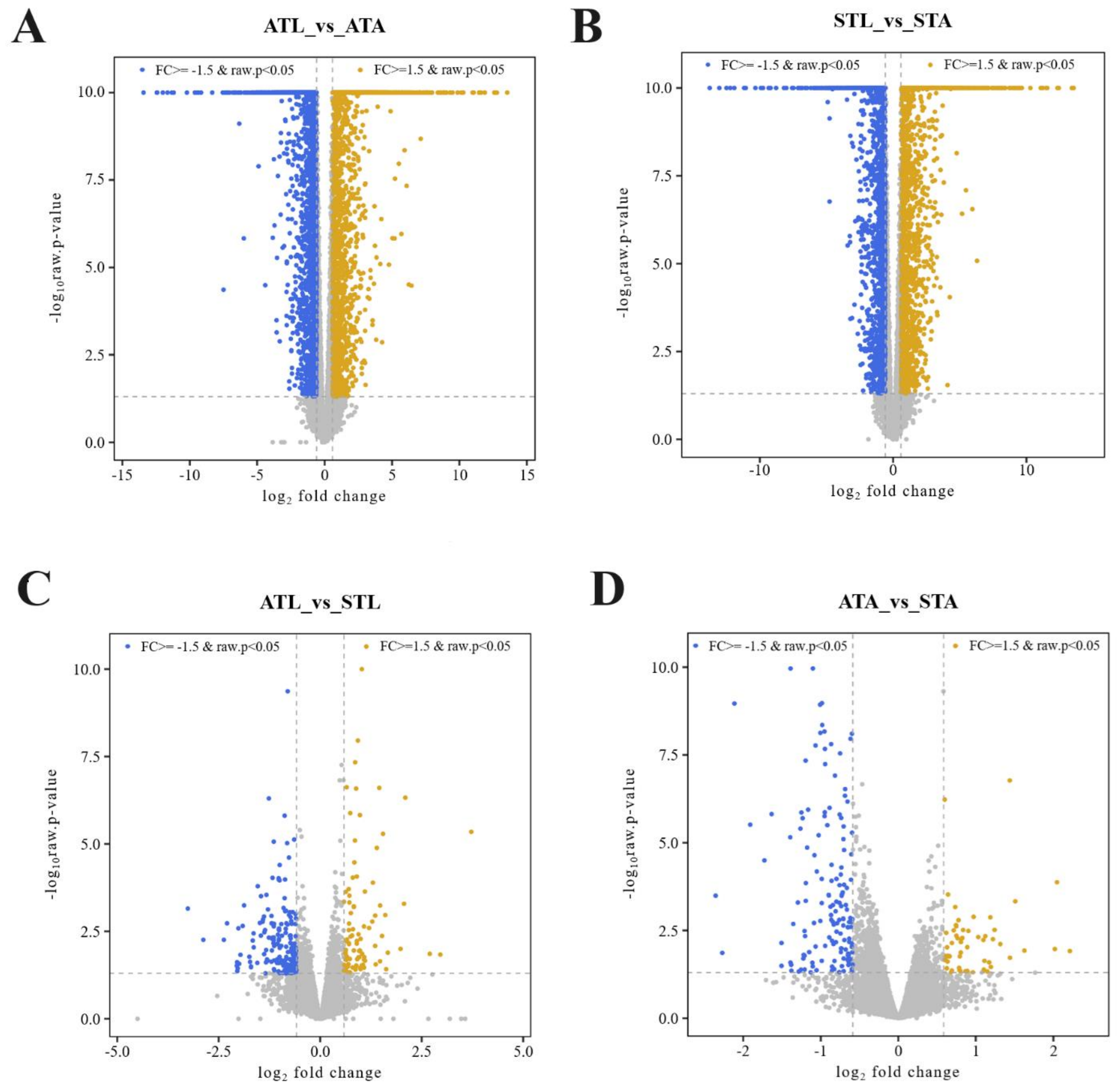

Figure S2: Comparative transcriptomic analyses of honeybee responses to spinetoram in honeybee larvae and adults. Volcano plots illustrate the differential expression of genes between four treatment comparisons: (A) ATL vs ATA, (B) STL vs STA, (C) ATL vs STL, (D) ATA vs STA. The data are presented as log2 fold changes against -log10 raw p-values. Gene expression differences are considered significant at a fold change (FC) of 1.5 and a raw p-value < 0.05, with upregulated genes shown in yellow and downregulated genes in blue. ATL, acetone-treated honeybee larvae; ATA, acetone-treated honeybee adults; STL, spinetoram-treated honeybee larvae; STA, spinetoram-treated honeybee adults.

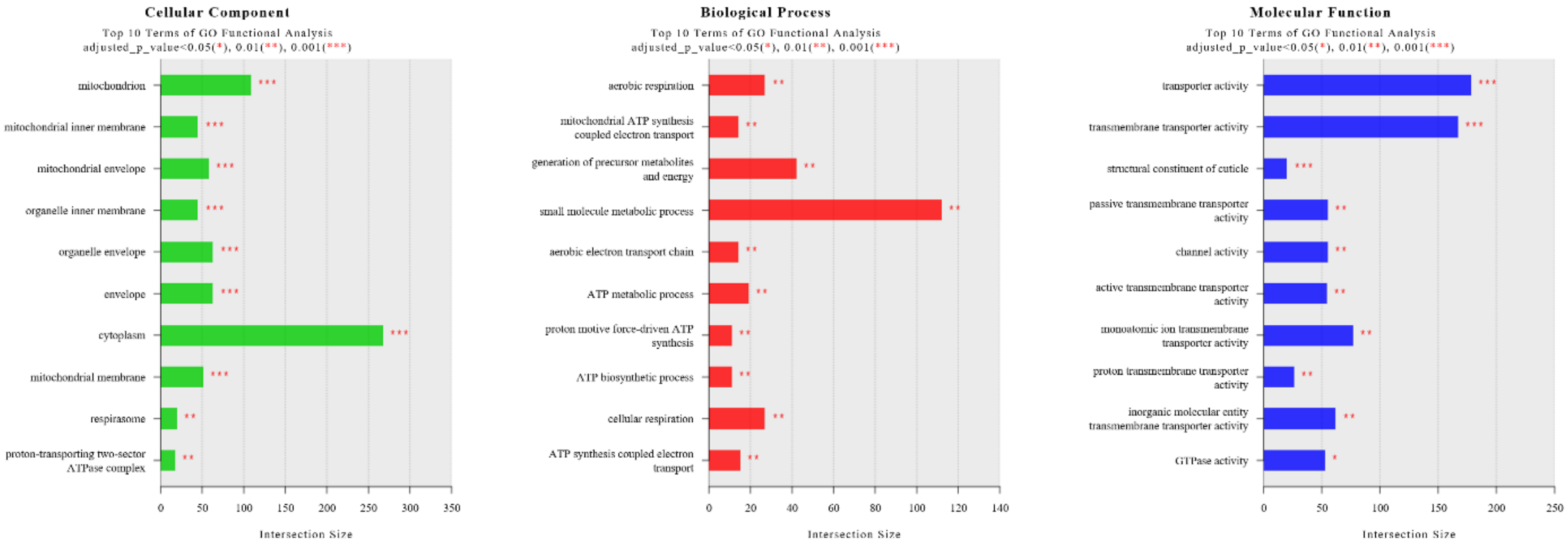

Figure S3: Top 10 terms of Gene Ontology (GO) functional analysis across three categories in ATA compared to ATL. Top terms in the category of Cellular Component, Biological Process and Molecular Function. Each bar represents the intersection size, with significance levels denoted by asterisks: a single asterisk (*) for p-values less than 0.05, double asterisks (**) for p-values less than 0.01, and triple asterisks (***) for p-values less than 0.001. ATL, acetone-treated honeybee larvae; ATA, acetone-treated honeybee adults.

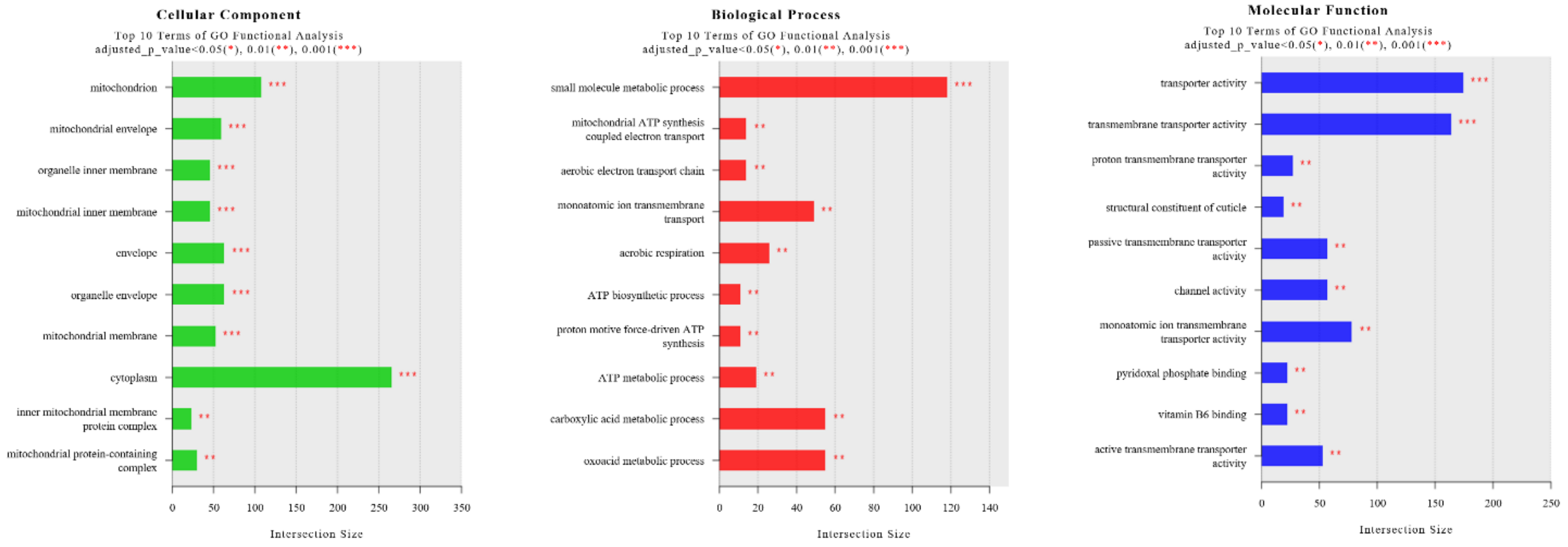

Figure S4: Top 10 terms of Gene Ontology (GO) functional analysis across three categories in STA compared to STL. Top terms in the category of Cellular Component, Biological Process and Molecular Function. Each bar represents the intersection size, with significance levels denoted by asterisks: a single asterisk (*) for p-values less than 0.05, double asterisks (**) for p-values less than 0.01, and triple asterisks (***) for p-values less than 0.001. STL, spinetoram-treated honeybee larvae; STA, spinetoram-treated honeybee adults.

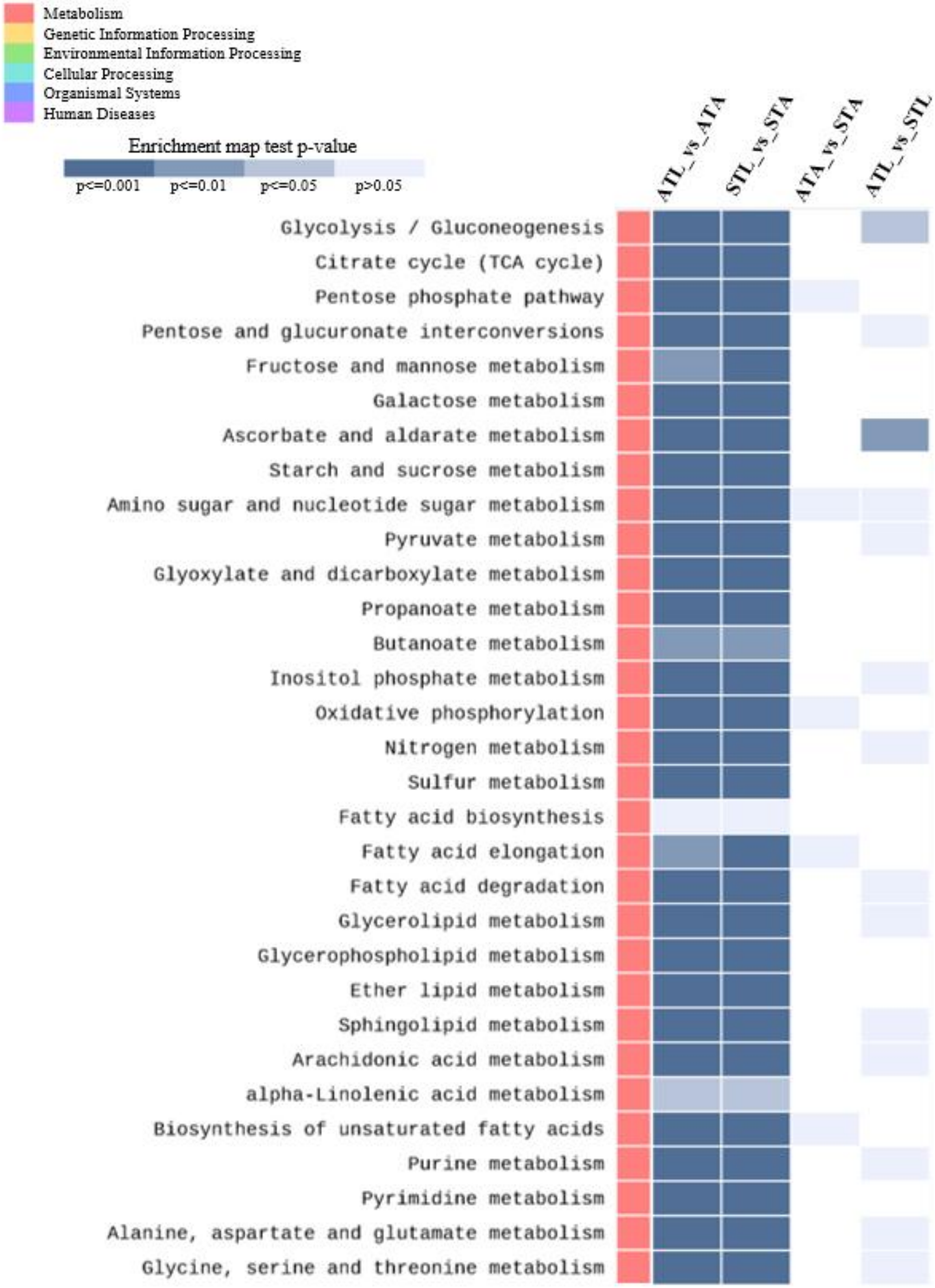

Metabolism
Genetic Information Processing
Environmental Information Processing
Cellular Processing
Organismal Systems
Human Diseases
Enrichment map test p-value
p<=0.001
p<=0.01
p<=0.05
p>0.05
ATL_vs_ATA
STL_vs_STA
ATA_vs_STA
ATL_vs_STL
Glycolysis / Gluconeogenesis
Citrate cycle (TCA cycle)
Pentose phosphate pathway
Pentose and glucuronate interconversions
Fructose and mannose metabolism
Galactose metabolism
Ascorbate and aldarate metabolism
Starch and sucrose metabolism
Amino sugar and nucleotide sugar metabolism
Pyruvate metabolism
Glyoxylate and dicarboxylate metabolism
Propanoate metabolism
Butanoate metabolism
Inositol phosphate metabolism
Oxidative phosphorylation
Nitrogen metabolism
Sulfur metabolism
Fatty acid biosynthesis
Fatty acid elongation
Fatty acid degradation
Glycerolipid metabolism
Glycerophospholipid metabolism
Ether lipid metabolism
Sphingolipid metabolism
Arachidonic acid metabolism
alpha-Linolenic acid metabolism
Biosynthesis of unsaturated fatty acids
Purine metabolism
Pyrimidine metabolism
Alanine, aspartate and glutamate metabolism
Glycine, serine and threonine metabolism

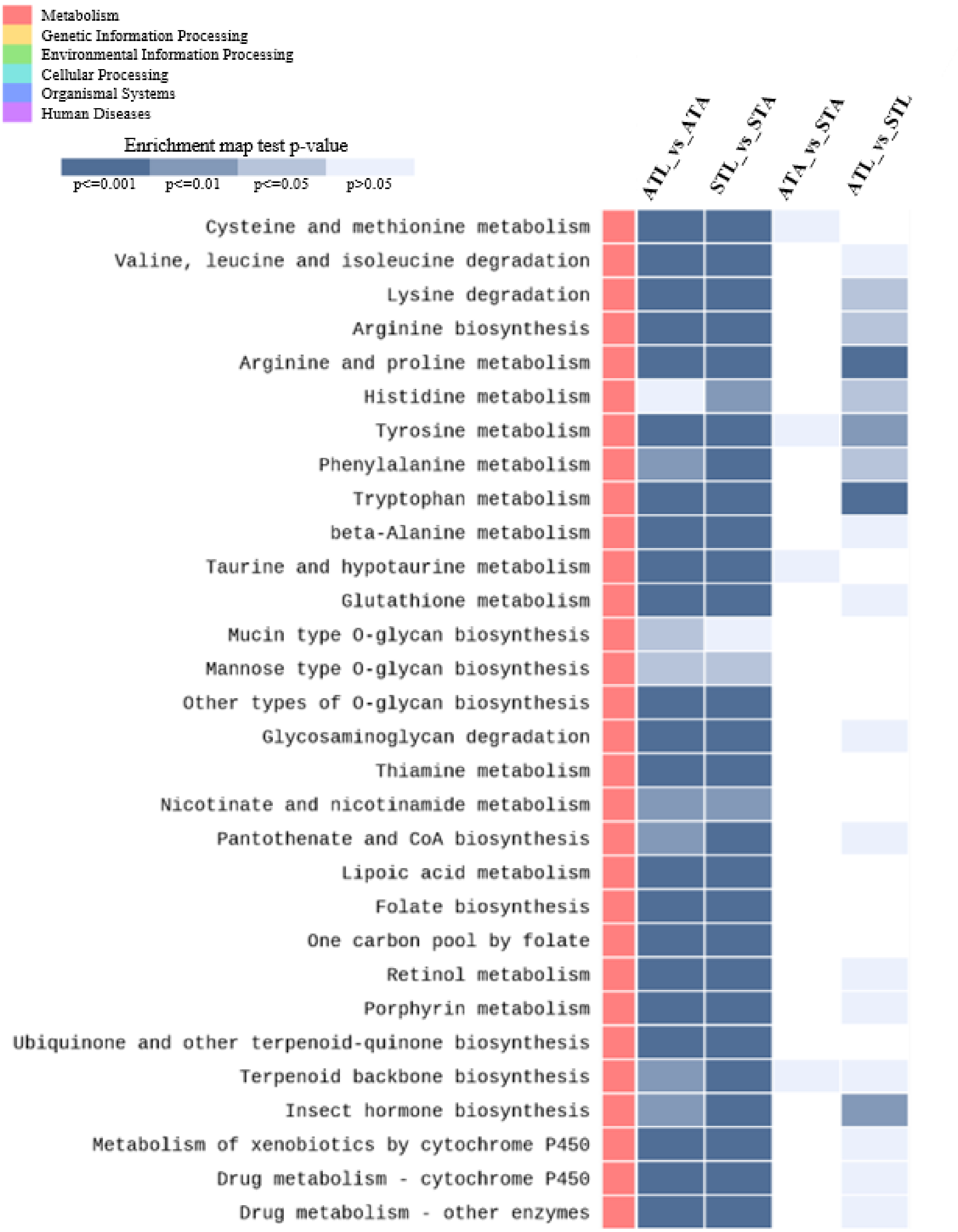

Metabolism
Genetic Information Processing
Environmental Information Processing
Cellular Processing
Organismal Systems
Human Diseases
Enrichment map test p-value
p<=0.001
p<=0.01
p<=0.05
p>0.05
ATL_vs_ATA
STL_vs_STA
ATA_vs_STA
ATL_vs_STL
Cysteine and methionine metabolism
Valine, leucine and isoleucine degradation
Lysine degradation
Arginine biosynthesis
Arginine and proline metabolism
Histidine metabolism
Tyrosine metabolism
Phenylalanine metabolism
Tryptophan metabolism
beta-Alanine metabolism
Taurine and hypotaurine metabolism
Glutathione metabolism
Mucin type O-glycan biosynthesis
Mannose type O-glycan biosynthesis
Other types of O-glycan biosynthesis
Glycosaminoglycan degradation
Thiamine metabolism
Nicotinate and nicotinamide metabolism
Pantothenate and CoA biosynthesis
Lipoic acid metabolism
Folate biosynthesis
One carbon pool by folate
Retinol metabolism
Porphyrin metabolism
Ubiquinone and other terpenoid-quinone biosynthesis
Terpenoid backbone biosynthesis
Insect hormone biosynthesis
Metabolism of xenobiotics by cytochrome P450
Drug metabolism - cytochrome P450
Drug metabolism - other enzymes

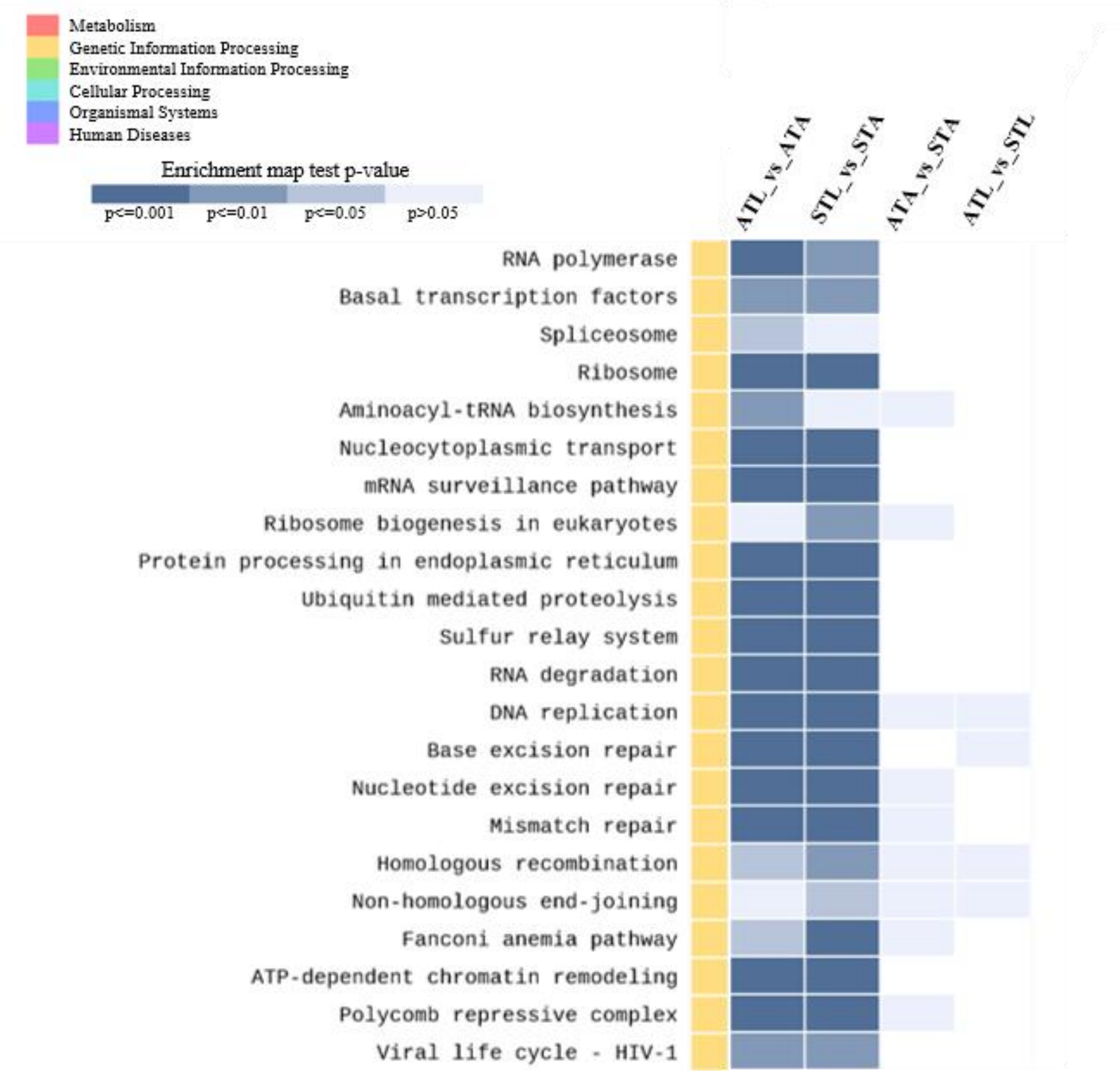
Metabolism
Genetic Information Processing
Environmental Information Processing
Cellular Processing
Organismal Systems
Human Diseases
Enrichment map test p-value
p<=0.001
p<=0.01
p<=0.05
p>0.05
ATL_vs_ATA
STL_vs_STA
ATA_vs_STA
ATL_vs_STL
RNA polymerase
Basal transcription factors
Spliceosome
Ribosome
Aminoacyl-tRNA biosynthesis
Nucleocytoplasmic transport
mRNA surveillance pathway
Ribosome biogenesis in eukaryotes
Protein processing in endoplasmic reticulum
Ubiquitin mediated proteolysis
Sulfur relay system
RNA degradation
DNA replication
Base excision repair
Nucleotide excision repair
Mismatch repair
Homologous recombination
Non-homologous end-joining
Fanconi anemia pathway
ATP-dependent chromatin remodeling
Polycomb repressive complex
Viral life cycle - HIV-1

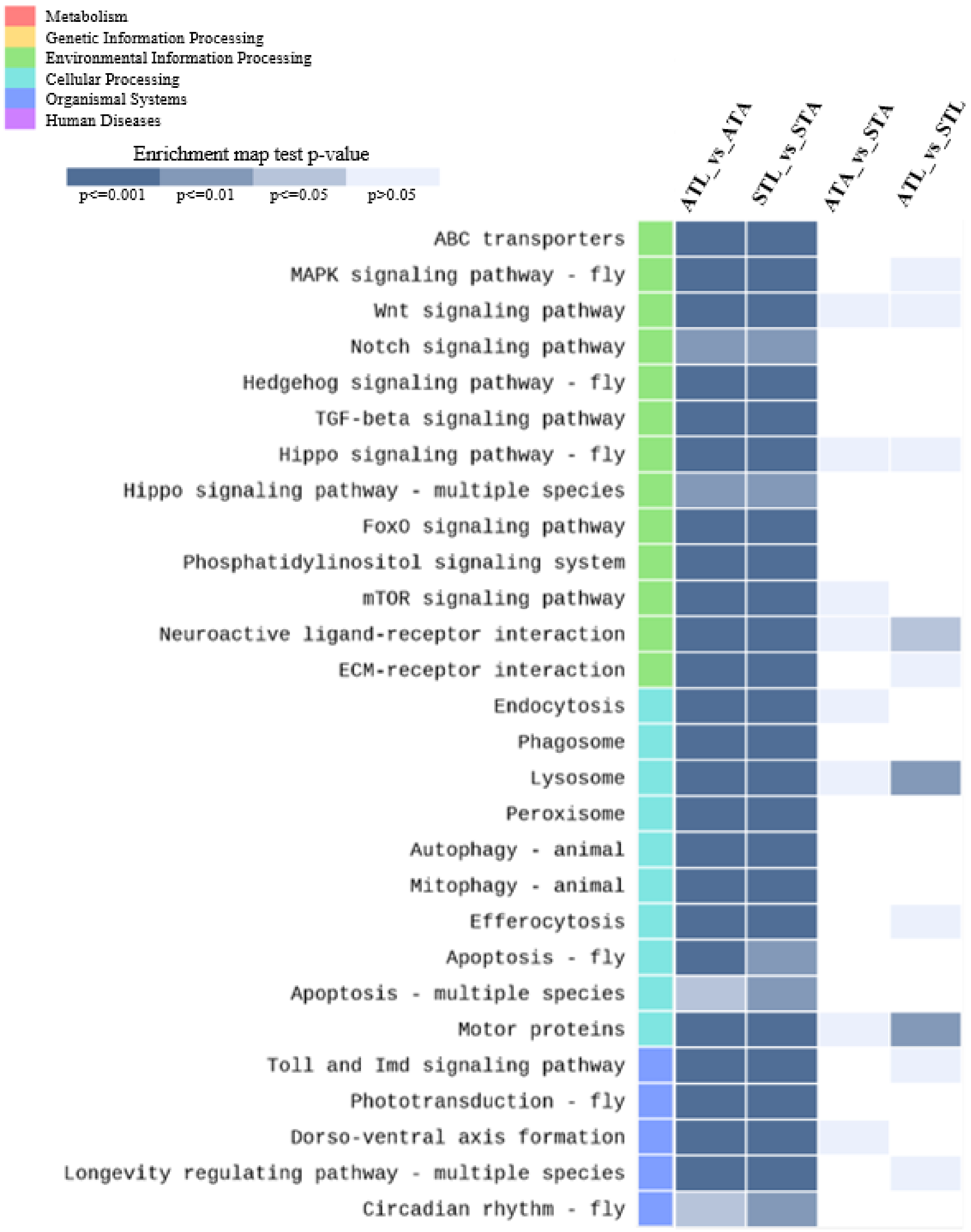

Figure S5: Comparative transcriptomic analysis of honeybee biological responses to spinetoram in larvae and adults. The heatmap shows the enrichment of various biological

pathways across four treatment comparisons: ATL vs. ATA, STL vs. STA, ATL vs. STL, and ATA vs. STA. Pathways are categorized into different biological processes, indicated by different colors: Metabolism (red), Genetic Information Processing (orange), Environmental Information Processing (green), Cellular Processing (blue), Organismal Systems (purple), and Human Diseases (pink). The intensity of the blue color in each cell represents the significance level (p-value) of the enrichment test, with darker shades indicating more significant enrichment ($p \leq 0.001$) and lighter shades indicating less significant enrichment ($p > 0.05$). ATL: acetone-treated honeybee larvae; ATA: acetone-treated honeybee adults; STL: spinetoram-treated honeybee larvae; STA: spinetoram-treated honeybee adults.

Table S1: List of DEGs for GO categories in ATA compared to ATL.

GO source	Term name (term_id)	Differentially Expressed Genes	P-Value
Biological Process	mitochondrial ATP synthesis coupled electron transport (GO:0042775)	**LOC725712, LOC412396, LOC725881, LOC409586, LOC551757, LOC726747, LOC409473, LOC100578782, LOC413340, LOC726316, LOC408837, Uqcr11, LOC551541, LOC552610**	$2.40x10^{-03}$
	aerobic respiration (GO:0009060)	**Pcl, LOC408734, LOC550686, LOC411014, LOC725712, LOC410396, LOC408446, LOC409549, LOC725566, LOC412396, LOC725881, LOC724264, LOC409586, LOC551757, LOC408950, LOC726747, LOC409473, LOC100578782, LOC413340, LOC552128, LOC726316, LOC409292, LOC410059, LOC408837, Uqcr11, LOC551541, LOC552610**	$2.40x10^{-03}$
	generation of precursor metabolites and energy (GO:0006091)	LOC550767, LOC409773, **Pcl, LOC408734, LOC552712, LOC550686, LOC552105, LOC107964213, LOC411014, LOC725325, LOC551154, LOC408818, LOC102655754, LOC725712, LOC410122, Tpi, LOC411576, LOC410396, LOC408446, LOC409549, LOC725566, LOC412396, LOC725881, LOC727599, LOC724264, LOC725797, LOC409586, LOC551757, LOC408950, LOC726747, LOC409473, LOC100578782, LOC413340, LOC552128, LOC726316, LOC409292, LOC410059, LOC408837, Uqcr11, LOC551541, LOC552610, CytC**	$3.09x10^{-03}$
	monoatomic ion transmembrane transport (GO:0034220)	nAChRa9, LOC408777, LOC551845, Tsf1, nAChRb2, LOC725645, LOC102654905, LOC411403, **LOC552410, nAChRa6, LOC409074, LOC726423, LOC412830, LOC552720, LOC413020, LOC552476, LOC100578899, LOC411036, LOC551883, LOC409055, LOC551093, LOC725219, LOC551961, nAChRa2, Para, pHCl, LOC408525, nAChRa8, nAChRb1, LOC410805, LOC551325, nAChRa7, LOC551454, Nmdar1, LOC102656594, LOC409114, Amel_8916, LOC551680, GluCl, LOC411732, LOC406124, LOC551766, LOC726268, ATP5G2, Hiscl1, LCCH3, LOC411664**	$6.71x10^{-03}$
	ATP metabolic process (GO:0046034)	**LOC551523, LOC551093, LOC551154, LOC408818, LOC102655754, LOC410122, Tpi, LOC411576, LOC409114,**	$4.17x10^{-03}$

		**LOC552699, LOC551861, LOC551766, LOC552682, LOC409236, LOC727483, LOC102654955, LOC726120, LOC410557, ATP5G2**	
	aerobic electron transport chain (GO:0019646)	**LOC725712, LOC412396, LOC725881, LOC409586, LOC551757, LOC726747, LOC409473, LOC100578782, LOC413340, LOC726316, LOC408837, Uqcr11, LOC551541, LOC552610**	$3.09x10^{-03}$
	proton motive force-driven ATP synthesis (GO:0015986)	**LOC409114, LOC552699, LOC551861, LOC551766, LOC552682, LOC409236, LOC727483, LOC102654955, LOC726120, LOC410557, ATP5G2**	$4.17x10^{-03}$
	ATP biosynthetic process (GO:0006754)	**LOC409114, LOC552699, LOC551861, LOC551766, LOC552682, LOC409236, LOC727483, LOC102654955, LOC726120, LOC410557, ATP5G2**	$4.17x10^{-03}$
	cellular respiration (GO:0045333)	**Pcl, LOC408734, LOC550686, LOC411014, LOC725712, LOC410396, LOC408446, LOC409549, LOC725566, LOC412396, LOC725881, LOC724264, LOC409586, LOC551757, LOC408950, LOC726747, LOC409473, LOC100578782, LOC413340, LOC552128, LOC726316, LOC409292, LOC410059, LOC408837, Uqcr11, LOC551541, LOC552610**	$4.51x10^{-03}$
	ATP synthesis coupled electron transport (GO:0042773)	**LOC725712, LOC412396, LOC725881, LOC724264, LOC409586, LOC551757, LOC726747, LOC409473, LOC100578782, LOC413340, LOC726316, LOC408837, Uqcr11, LOC551541, LOC552610**	$6.71x10^{-03}$
Cellular Component	mitochondrion (GO:0005739)	LOC100576847, LOC412308, LOC102655740, LOC410410, LOC409667, LOC411790, LOC408539, LOC725527, LOC410325, LOC551710, **LOC725105, LOC551492, LOC413878, LOC408734, LOC725240, LOC724988, LOC552635, LOC413879, LOC413228, LOC551523, LOC408569, LOC412796, LOC411304, LOC408352, LOC411142, LOC552802, LOC552644, LOC552526, LOC551337, LOC726498, LOC725705, LOC100577341, LOC413774, LOC412082, LOC550915, LOC551329, LOC725854, LOC413438, LOC552354, LOC413762, LOC412409, LOC724790, LOC410856, LOC411351, LOC413186, LOC410612, LOC411448, Coq7, LOC408968, LOC408548, LOC102654007, LOC551939, LOC727014, LOC413781, LOC726731, LOC726061, LOC726617,**	$7.95x10^{-13}$

	**LOC412984, LOC551158, LOC411924, LOC410791, LOC551325, LOC725712, LOC552152, LOC100576960, LOC727026, LOC726239, LOC408511, LOC726902, LOC100302106, Ant, LOC408446, LOC725566, LOC725315, Ndufb2, LOC412396, Cox6b1, LOC552009, LOC551660, LOC725881, LOC551042, LOC727599, LOC724264, LOC725797, LOC409586, LOC551757, LOC724827, LOC411677, LOC551861, LOC726747, Ndufs5, LOC409473, LOC410022, LOC413517, LOC100578782, LOC413340, LOC552128, LOC725253, LOC727483, LOC102654955, LOC726120, LOC726316, LOC409292, LOC410557, LOC408837, Uqcr11, LOC551541, LOC552610, CytC**	
mitochondrial inner membrane (GO:0005743)	LOC411790, LOC725527, **LOC725105, LOC408734, LOC408569, LOC551337, LOC726498, LOC725705, LOC412082, LOC412409, LOC413186, Coq7, LOC408968, LOC408548, LOC102654007, LOC413781, LOC725712, LOC726902, Ant, LOC725566, Ndufb2, LOC412396, LOC552009, LOC725881, LOC551042, LOC727599, LOC724264, LOC551757, LOC411677, LOC551861, LOC726747, LOC409473, LOC413517, LOC100578782, LOC413340, LOC725253, LOC727483, LOC102654955, LOC726120, LOC726316, LOC410557, LOC408837, Uqcr11, LOC551541, LOC552610**	3.40×10^{-06}
organelle inner membrane (GO:0019866)	LOC411790, LOC725527, **LOC725105, LOC408734, LOC408569, LOC551337, LOC726498, LOC725705, LOC412082, LOC412409, LOC413186, Coq7, LOC408968, LOC408548, LOC102654007, LOC413781, LOC725712, LOC726902, Ant, LOC725566, Ndufb2, LOC412396, LOC552009, LOC725881, LOC551042, LOC727599, LOC724264, LOC551757, LOC411677, LOC551861, LOC726747, LOC409473, LOC413517, LOC100578782, LOC413340, LOC725253, LOC727483, LOC102654955, LOC726120, LOC726316, LOC410557, LOC408837, Uqcr11, LOC551541, LOC552610**	3.40×10^{-06}
mitochondrial envelope (GO:0005740)	LOC102655740, LOC409667, LOC411790, LOC408539, LOC725527, **LOC725105, LOC408734, LOC551523, LOC408569, LOC411142,**	3.40×10^{-06}

	**LOC551337, LOC726498, LOC725705, LOC412082, LOC550915, LOC552354, LOC412409, LOC413186, Coq7, LOC408968, LOC408548, LOC102654007, LOC413781, LOC726061, LOC410791, LOC551325, LOC725712, LOC727026, LOC408511, LOC726902, Ant, LOC725566, Ndufb2, LOC412396, LOC552009, LOC725881, LOC551042, LOC727599, LOC724264, LOC551757, LOC411677, LOC551861, LOC726747, LOC409473, LOC413517, LOC100578782, LOC413340, LOC725253, LOC727483, LOC102654955, LOC726120, LOC726316, LOC410557, LOC408837, Uqcr11, LOC551541, LOC552610, CytC**	
organelle envelope (GO:0031967)	LOC102655740, LOC409667, LOC411918, LOC413675, LOC411790, LOC412464, LOC408539, LOC725527, LOC410343, LOC410972, **LOC725105, LOC408734, LOC551523, LOC408569, LOC411142, LOC551337, LOC726498, LOC725705, LOC412082, LOC550915, LOC552354, LOC412409, LOC413186, Coq7, LOC408968, LOC408548, LOC102654007, LOC413781, LOC726061, LOC410791, LOC551325, LOC725712, LOC727026, LOC408511, LOC726902, Ant, LOC725566, Ndufb2, LOC412396, LOC552009, LOC725881, LOC551042, LOC727599, LOC724264, LOC551757, LOC411677, LOC551861, LOC726747, LOC409473, LOC413517, LOC100578782, LOC413340, LOC725253, LOC727483, LOC102654955, LOC726120, LOC726316, LOC410557, LOC408837, Uqcr11, LOC551541, LOC552610, CytC**	4.71×10^{-06}
envelope (GO:0031975)	LOC102655740, LOC409667, LOC411918, LOC413675, LOC411790, LOC412464, LOC408539, LOC725527, LOC410343, LOC410972, **LOC725105, LOC408734, LOC551523, LOC408569, LOC411142, LOC551337, LOC726498, LOC725705, LOC412082, LOC550915, LOC552354, LOC412409, LOC413186, Coq7, LOC408968, LOC408548, LOC102654007, LOC413781, LOC726061, LOC410791, LOC551325, LOC725712, LOC727026, LOC408511, LOC726902, Ant, LOC725566, Ndufb2, LOC412396, LOC552009, LOC725881, LOC551042, LOC727599, LOC724264, LOC551757, LOC411677, LOC551861, LOC726747, LOC409473, LOC413517,**	4.71×10^{-06}

	**LOC100578782, LOC413340, LOC725253, LOC727483, LOC102654955, LOC726120, LOC726316, LOC410557, LOC408837, Uqcr11, LOC551541, LOC552610, CytC**	
cytoplasm (GO:0005737)	Hex110, LOC410994, LOC552313, LOC409435, LOC408987, LOC409520, LOC100576847, LOC412308, LOC408650, LOC412544, LOC411147, LOC726887, PRF, LOC102655740, LOC409023, LOC410410, LOC724947, LOC409667, LOC411378, LOC550827, LOC409576, LOC412097, nanos, LOC411088, LOC551986, LOC725789, LOC412823, LOC413742, LOC409485, LOC409809, LOC413613, Ripk5, LOC408808, LOC551282, LOC552560, LOC551466, Arp1, LOC409313, LOC100576667, LOC409543, LOC411790, EF1a-F2, LOC724741, LOC550716, LOC408388, LOC412608, LOC408301, LOC100576432, LOC410808, LOC726419, LOC726546, LOC408539, LOC725527, LOC725018, LOC408687, LOC551578, LOC552766, LOC551438, LOC550798, LOC410325, LOC413614, LOC411799, LOC725817, LOC411765, LOC102653815, LOC410869, LOC552363, LOC412511, LOC408751, LOC411970, LOC411533, LOC551802, LOC552346, LOC409301, LOC551472, Gpdh, LOC550694, LOC409321, LOC551771, LOC551420, LOC413299, LOC410486, LOC411870, LOC552140, LOC551710, LOC552118, LOC551436, LOC724505, LOC550673, LOC551408, LOC409718, LOC410614, LOC412169, LOC410533, LOC551184, LOC726132, LOC552540, **LOC552256, LOC726609, LOC725105, LOC102656491, LOC408391, LOC551492, LOC102656006, LOC410922, LOC413878, LOC725742, LOC726205, LOC409015, LOC552001, LOC550700, LOC724188, LOC727300, LOC408734, LOC412710, LOC412119, LOC409934, LOC409487, LOC551477, LOC551580, LOC102655898, LOC726815, LOC100577378, LOC725240, LOC411654, LOC552533, LOC726894, LOC724988, LOC552635, LOC413879, LOC550686, LOC725194, LOC726176, LOC413228, LOC551523, LOC408569, LOC102655877, LOC412796, LOC107964213, LOC413601, LOC552377, LOC411304, LOC408352, LOC552705, LOC100579024,**	$6.89x10^{-05}$

	**LOC552014, LOC413889, LOC409905, LOC724496, LOC411142, LOC552802, LOC725680, LOC411459, LOC409055, LOC725977, LOC552181, LOC552644, LOC552526, LOC726164, LOC551337, LOC726498, LOC724241, LOC551354, LOC551961, LOC551978, LOC551216, LOC725705, LOC100577341, LOC552745, LOC413774, LOC412082, LOC550915, LOC551329, LOC725854, LOC413438, LOC552354, LOC413762, LOC412409, LOC724790, LOC100578006, LOC410856, LOC411351, LOC413186, LOC410612, LOC552519, LOC411448, Coq7, LOC408968, LOC408548, LOC102654007, LOC551939, LOC727014, LOC410996, LOC413781, LOC726731, LOC726061, Cry2, Syt1, LOC726617, LOC100577548, LOC412984, LOC412886, LOC100576735, LOC551158, LOC102655754, LOC411924, LOC410791, LOC551325, LOC409299, LOC725712, LOC552152, LOC413139, LOC100576960, LOC727026, LOC726239, LOC408511, LOC726902, LOC102654353, Glob1, LOC100302106, Ant, LOC408446, LOC725566, LOC725315, Ndufb2, LOC412396, Cox6b1, LOC552009, LOC408614, LOC551660, LOC551631, TpnI, LOC725881, LOC551042, LOC410371, LOC727599, LOC724264, LOC725797, LOC409586, LOC551757, LOC724827, LOC411677, LOC551861, LOC726747, Ef-1a-f1, Ndufs5, LOC409473, LOC410022, LOC413517, Gycbeta1, LOC100578782, LOC413340, LOC552128, LOC725253, LOC727483, LOC102654955, LOC726120, TpnT, LOC726316, LOC409292, LOC410557, LOC408837, sGC-alpha1, Uqcr11, LOC551541, LOC552610, LOC107964319, CytC**	
mitochondrial membrane (GO:0031966)	LOC102655740, LOC411790, LOC408539, LOC725527, **LOC725105, LOC408734, LOC408569, LOC411142, LOC551337, LOC726498, LOC725705, LOC412082, LOC550915, LOC412409, LOC413186, Coq7, LOC408968, LOC408548, LOC102654007, LOC413781, LOC410791, LOC551325, LOC725712, LOC726902, Ant, LOC725566, Ndufb2, LOC412396, LOC552009, LOC725881, LOC551042, LOC727599, LOC724264, LOC551757, LOC411677,**	8.24×10^{-05}

		**LOC551861, LOC726747, LOC409473, LOC413517, LOC100578782, LOC413340, LOC725253, LOC727483, LOC102654955, LOC726120, LOC726316, LOC410557, LOC408837, Uqcr11, LOC551541, LOC552610**	
	respirasome (GO:0070469)	LOC100576847, LOC725527, **LOC725712, LOC409549, Ndufb2, LOC412396, Cox6b1, LOC551042, LOC727599, LOC551757, LOC726747, LOC409473, LOC100578782, LOC413340, LOC726316, LOC408837, Uqcr11, LOC551541, LOC552610, CytC**	6.93×10^{-03}
	respiratory chain complex (GO:0098803)	LOC100576847, **LOC725712, LOC409549, Ndufb2, LOC412396, Cox6b1, LOC551042, LOC551757, LOC726747, LOC100578782, LOC413340, LOC726316, LOC408837, Uqcr11, LOC551541, LOC552610**	7.72×10^{-03}
Molecular Function	transporter activity (GO:0005215)	Vhdl, LOC725922, LOC725462, nAChRa9, LOC410967, LOC726412, LOC412764, LOC100578810, LOC726762, LOC408777, LOC727346, LOC414051, LOC551845, LOC408467, LOC100578207, LOC408634, LOC102655740, LOC411955, nAChRb2, LOC409467, LOC413259, LOC551250, LOC408478, LOC408828, LOC550918, LOC551553, LOC551263, LOC408517, LOC410578, LOC552592, LOC411935, LOC410915, LOC413739, LOC724952, LOC412430, LOC411790, LOC408456, LOC726724, LOC552144, LOC411997, LOC408539, LOC412056, LOC725527, LOC552653, LOC551806, LOC552640, LOC409424, LOC412741, LOC411807, LOC552552, LOC726513, LOC411403, LOC551894, LOC410533, LOC410062, **LOC552708, LOC413689, LOC726721, LOC409865, LOC552410, LOC552001, nAChRa6, LOC413934, LOC552744, LOC409074, LOC409666, LOC552704, LOC409899, LOC726423, LOC412830, LOC552720, LOC410997, LOC413020, LOC409897, LOC552476, LOC100578899, LOC411052, LOC410626, LOC408402, LOC552727, LOC411036, LOC551883, LOC409055, LOC551093, LOC551337, LOC412431, LOC410566, LOC551936, LOC409932, LOC413959, LOC725219, LOC551961, LOC552149, nAChRa2, LOC409565, LOC550915, LOC409192, Para, LOC411764, LOC409056, pHCl, LOC410612, LOC100578218, LOC410278,**	2.51×10^{-07}

		**LOC409715, LOC408525, nAChRa8, LOC409636, nAChRb1, LOC410805, LOC551167, LOC409924, LOC410803, LOC413816, LOC410791, LOC551325, LOC409919, nAChRa7, LOC551454, LOC100578557, LOC408769, LOC410788, LOC409073, LOC726793, Nmdar1, Gat-a, LOC551782, LOC409114, Ant, LOC102653800, LOC725315, LOC412396, LOC552792, LOC551660, Amel_8916, LOC551680, LOC725881, LOC410371, GluCl, LOC724264, LOC411732, LOC408909, LOC406124, LOC552699, LOC409646, LOC408992, LOC412007, LOC724827, LOC551861, Gat-1B, LOC551766, LOC726747, LOC552682, LOC413517, Eaat-2, LOC409236, LOC411411, LOC727483, LOC102654955, LOC726120, LOC726316, LOC412220, LOC412777, LOC410557, LOC408837, ATP5G2, Hiscl1, LCCH3, LOC551604, Uqcr11, LOC725346, LOC551541, LOC552610, LOC408696, LOC725165, LOC412925, Vg, Melt**	
	transmembrane transporter activity (GO:0022857)	LOC725922, LOC725462, nAChRa9, LOC410967, LOC726412, LOC412764, LOC100578810, LOC726762, LOC408777, LOC727346, LOC414051, LOC551845, LOC408467, LOC100578207, LOC408634, LOC102655740, nAChRb2, LOC409467, LOC413259, LOC408478, LOC408828, LOC550918, LOC551553, LOC551263, LOC408517, LOC410578, LOC552592, LOC411935, LOC410915, LOC413739, LOC724952, LOC412430, LOC411790, LOC408456, LOC726724, LOC552144, LOC411997, LOC408539, LOC412056, LOC725527, LOC552653, LOC551806, LOC552640, LOC409424, LOC412741, LOC411807, LOC552552, LOC726513, LOC411403, LOC551894, LOC410062, **LOC552708, LOC413689, LOC726721, LOC552410, LOC552001, nAChRa6, LOC413934, LOC552744, LOC409074, LOC409666, LOC552704, LOC409899, LOC726423, LOC412830, LOC552720, LOC410997, LOC413020, LOC409897, LOC552476, LOC100578899, LOC411052, LOC410626, LOC408402, LOC552727, LOC411036, LOC551883, LOC409055, LOC551093, LOC551337, LOC412431, LOC410566, LOC551936, LOC409932, LOC413959, LOC725219, LOC551961, LOC552149, nAChRa2,**	$1.27x10^{-06}$

	**LOC409565, LOC550915, Para, LOC411764, LOC409056, pHCl, LOC410612, LOC100578218, LOC410278, LOC409715, LOC408525, nAChRa8, nAChRb1, LOC410805, LOC551167, LOC409924, LOC410803, LOC413816, LOC410791, LOC551325, LOC409919, nAChRa7, LOC551454, LOC100578557, LOC408769, LOC410788, LOC409073, Nmdar1, Gat-a, LOC551782, LOC409114, Ant, LOC102653800, LOC725315, LOC412396, LOC552792, LOC551660, Amel_8916, LOC551680, LOC725881, LOC410371, GluCl, LOC724264, LOC411732, LOC408909, LOC406124, LOC552699, LOC409646, LOC412007, LOC724827, LOC551861, Gat-1B, LOC551766, LOC726747, LOC552682, LOC413517, Eaat-2, LOC409236, LOC411411, LOC727483, LOC102654955, LOC726120, LOC726316, LOC412220, LOC412777, LOC410557, LOC408837, ATP5G2, Hiscl1, LCCH3, LOC551604, Uqcr11, LOC725346, LOC551541, LOC552610, LOC725165, LOC412925, Melt**	
structural constituent of cuticle (GO:0042302)	CPR21, CPR1, LOC102654371, CPR13, CPR16, LOC102653832, CPR4, CPR2, **CPR17, CPR11, LOC107964828, LOC726725, CPR3, CPR27, LOC727578, CPR5, CPR6, LOC724624, CPR19, CPR14**	$3.49x10^{-04}$
monoatomic ion transmembrane transporter activity (GO:0015075)	nAChRa9, LOC408777, LOC727346, LOC551845, LOC408467, LOC100578207, nAChRb2, LOC726724, LOC408539, LOC725527, LOC411807, LOC552552, LOC411403, LOC551894, **LOC413689, LOC552410, nAChRa6, LOC409074, LOC552704, LOC726423, LOC412830, LOC552720, LOC413020, LOC552476, LOC100578899, LOC411036, LOC551883, LOC409055, LOC551093, LOC725219, LOC551961, nAChRa2, LOC550915, Para, LOC411764, pHCl, LOC409715, LOC408525, nAChRa8, nAChRb1, LOC410805, LOC410803, LOC551325, nAChRa7, LOC551454, LOC410788, Nmdar1, LOC551782, LOC409114, LOC102653800, LOC412396, LOC552792, Amel_8916, LOC551680, LOC410371, GluCl, LOC411732, LOC406124, LOC552699, LOC551861, LOC551766, LOC726747, LOC552682, LOC409236, LOC727483, LOC102654955, LOC726120,**	$3.28x10^{-03}$

	**LOC726316, LOC412777, LOC410557, LOC408837, ATP5G2, Hiscl1, LCCH3, Uqcr11, LOC551541, LOC552610**	
passive transmembrane transporter activity (GO:0022803)	nAChRa9, LOC408777, LOC727346, LOC551845, LOC408467, LOC100578207, nAChRb2, LOC413259, LOC410915, LOC726724, LOC551806, LOC552640, LOC552552, LOC551894, **nAChRa6, LOC726423, LOC412830, LOC413020, LOC100578899, LOC411036, LOC551883, LOC725219, LOC552149, nAChRa2, Para, LOC411764, pHCl, LOC408525, nAChRa8, nAChRb1, LOC410805, LOC551325, nAChRa7, LOC551454, LOC410788, Nmdar1, LOC551782, LOC409114, LOC102653800, LOC552792, Amel_8916, LOC551680, GluCl, LOC411732, LOC406124, LOC552699, LOC551766, LOC552682, LOC409236, LOC727483, Hiscl1, LCCH3, LOC551604, LOC725165, Melt**	$3.28x10^{-03}$
proton transmembrane transporter activity (GO:0015078)	LOC725527, **LOC552410, LOC409074, LOC552720, LOC552476, LOC409055, LOC551093, LOC551961, LOC409114, LOC412396, LOC552699, LOC551861, LOC551766, LOC726747, LOC552682, LOC409236, LOC727483, LOC102654955, LOC726120, LOC726316, LOC410557, LOC408837, ATP5G2, Uqcr11, LOC551541, LOC552610**	$3.28x10^{-03}$
active transmembrane transporter activity (GO:0022804)	LOC410967, LOC414051, LOC550918, LOC410578, LOC724952, LOC411997, LOC725527, LOC412741, LOC726513, **LOC413689, LOC726721, LOC552410, LOC413934, LOC552744, LOC409074, LOC409666, LOC552720, LOC409897, LOC552476, LOC409055, LOC551093, LOC551337, LOC413959, LOC551961, LOC409056, LOC100578218, LOC409715, LOC551167, LOC413816, LOC409919, LOC408769, LOC409073, Gat-a, Ant, LOC725315, LOC412396, LOC551660, LOC725881, LOC410371, LOC724264, LOC408909, LOC724827, Gat-1B, LOC726747, LOC413517, Eaat-2, LOC411411, LOC726316, LOC412777, LOC408837, Uqcr11, LOC725346, LOC551541, LOC552610**	$3.28x10^{-03}$
channel activity (GO:0015267)	nAChRa9, LOC408777, LOC727346, LOC551845, LOC408467, LOC100578207, nAChRb2, LOC413259, LOC410915, LOC726724, LOC551806, LOC552640, LOC552552, LOC551894, **nAChRa6,**	$3.28x10^{-03}$

		**LOC726423, LOC412830, LOC413020, LOC100578899, LOC411036, LOC551883, LOC725219, LOC552149, nAChRa2, Para, LOC411764, pHCl, LOC408525, nAChRa8, nAChRb1, LOC410805, LOC551325, nAChRa7, LOC551454, LOC410788, Nmdar1, LOC551782, LOC409114, LOC102653800, LOC552792, Amel_8916, LOC551680, GluCl, LOC411732, LOC406124, LOC552699, LOC551766, LOC552682, LOC409236, LOC727483, Hiscl1, LCCH3, LOC551604, LOC725165, Melt**	
	inorganic molecular entity transmembrane transporter activity (GO:0015318)	LOC408777, LOC551845, LOC408467, LOC100578207, LOC724952, LOC725527, LOC552640, LOC411807, LOC411403, **LOC413689, LOC552410, LOC413934, LOC409074, LOC552704, LOC412830, LOC552720, LOC413020, LOC552476, LOC100578899, LOC411036, LOC551883, LOC409055, LOC551093, LOC725219, LOC551961, Para, LOC411764, LOC409056, pHCl, LOC409715, LOC410805, LOC413816, LOC551454, LOC551782, LOC409114, LOC412396, LOC551680, LOC410371, GluCl, LOC411732, LOC406124, LOC552699, LOC551861, LOC551766, LOC726747, LOC552682, LOC413517, LOC409236, LOC727483, LOC102654955, LOC726120, LOC726316, LOC412777, LOC410557, LOC408837, ATP5G2, Hiscl1, LCCH3, LOC551604, Uqcr11, LOC551541, LOC552610**	3.76x10^{-03}
	GTPase activity (GO:0003924)	LOC410994, LOC409633, LOC411704, LOC726887, LOC411226, LOC552138, LOC410906, LOC550827, LOC411642, LOC552042, LOC100577870, LOC410280, LOC724594, LOC412030, EF1a-F2, LOC408388, LOC413827, LOC102656587, LOC409342, LOC552766, LOC413614, LOC552625, LOC100577580, LOC102654987, LOC409863, LOC411134, LOC726027, LOC724366, LOC410614, LOC409126, LOC409481, **LOC411085, LOC408556, LOC552730, LOC107965449, LOC411252, LOC725337, LOC408522, LOC550886, LOC100578704, LOC408328, LOC724762, LOC413438, LOC411351, LOC413034, LOC410996, LOC100577548, LOC552419, LOC412886, LOC725453, LOC410943, Ef-1a-f1, LOC408971**	1.01x10^{-02}

The table presents the Gene ontology source, term name (term id), associated DEGs, and p-values. DEGs shown in bold are upregulated, while those in normal text are downregulated in acetone-treated honeybee adults (ATA) compared to acetone-treated honeybee larvae (ATL). The DEGs in each GO term are displayed from highest downregulation to highest upregulation.

Table S2: List of DEGs for GO categories in STA compared to STL.

GO source	Term_name (term_id)	Differentially Expressed Genes	P-Value
Biological Process	monoatomic ion transmembrane transport (GO:0034220)	nAChRa9, LOC408777, LOC551845, Tsf1, nAChRb2, LOC408841, LOC411403, LOC725645, **LOC552410, LOC102654379, Amih, LOC726423, LOC552720, LOC409074, LOC100577376, LOC411036, LOC409055, LOC551883, LOC552476, LOC551093, LOC412830, LOC408525, nAChRa2, LOC100578899, Para, nAChRb1, LOC725219, LOC551961, nAChRa8, pHCl, nAChRa6, LOC551325, LOC410805, LOC551454, nAChRa7, Nmdar1, LOC102656594, LOC409114, GluCl, Amel_8916, LOC406124, LOC411732, LOC551766, LOC551680, LOC726268, ATP5G2, LCCH3, Hiscl1, LOC411664**	4.04x10^{-03}
	mitochondrial ATP synthesis coupled electron transport (GO:0042775)	**LOC725712, LOC412396, LOC725881, LOC100578782, LOC409586, LOC551757, LOC413340, LOC409473, LOC726747, LOC408837, LOC726316, Uqcr11, LOC552610, LOC551541**	1.96x10^{-03}
	aerobic electron transport chain (GO:0019646)	**LOC725712, LOC412396, LOC725881, LOC100578782, LOC409586, LOC551757, LOC413340, LOC409473, LOC726747, LOC408837, LOC726316, Uqcr11, LOC552610, LOC551541**	4.04x10^{-03}
	ATP metabolic process (GO:0046034)	**LOC551523, LOC551093, LOC551154, LOC408818, Tpi, LOC410122, LOC102655754, LOC411576, LOC409114, LOC552699, LOC552682, LOC551766, LOC409236, LOC102654955, LOC551861, LOC410557, LOC726120, ATP5G2, LOC727483**	4.86x10^{-03}
	aerobic respiration (GO:0009060)	**LOC550686, LOC408734, LOC411014, LOC725712, LOC725566, LOC409549, LOC410396, LOC412396, LOC408446, LOC725881, LOC100578782, LOC409586, LOC724264, LOC408950, LOC551757, LOC413340, LOC409473, LOC726747, LOC552128, LOC408837, LOC409292, LOC410059, LOC726316, Uqcr11, LOC552610, LOC551541**	4.67x10^{-03}

	proton motive force-driven ATP synthesis (GO:0015986)	**LOC409114, LOC552699, LOC552682, LOC551766, LOC409236, LOC102654955, LOC551861, LOC410557, LOC726120, ATP5G2, LOC727483**	$4.81x10^{-03}$
	ATP biosynthetic process (GO:0006754)	**LOC409114, LOC552699, LOC552682, LOC551766, LOC409236, LOC102654955, LOC551861, LOC410557, LOC726120, ATP5G2, LOC727483**	$4.81x10^{-03}$
	small molecule metabolic process (GO:0044281)	LOC550828, LOC408650, LOC411796, LOC412467, LOC410422, LOC409066, LOC408859, LOC409023, LOC551986, LOC412876, LOC551282, LOC412619, LOC551143, LOC724909, LOC409179, LOC550767, LOC413789, LOC551448, LOC410325, LOC409444, LOC411088, LOC551835, LOC725031, LOC412460, LOC413702, LOC409846, LOC408509, LOC414008, LOC412815, GlnS, LOC725018, LOC552382, LOC409773, LOC725817, LOC551593, LOC551447, LOC552823, **LOC726205, LOC725105, LOC412234, LOC412796, LOC551523, LOC411189, LOC102656439, LOC413697, LOC551948, LOC413763, LOC550686, LOC727293, LOC410583, LOC409487, LOC552014, LOC552086, LOC725816, LOC413601, LOC100577378, LOC552644, LOC551676, LOC551093, LOC412069, LOC411014, LOC726262, LOC410639, LOC725325, LOC411771, LOC726754, LOC551154, LOC412082, LOC410539, LOC409963, LOC411633, LOC408441, LOC408474, LOC409063, LOC550885, LOC724791, LOC410948, LOC725400, LOC100578936, LOC410120, LOC410612, Coq7, LOC408818, LOC411916, LOC552316, Tpi, LOC410638, LOC409299, LOC410122, Ac3, LOC410828, LOC725204, LOC102655754, LOC411576, LOC725255, LOC409114, LOC552699, LOC551631, LOC551103, LOC412541, LOC552682, Gycbeta1, LOC408950, LOC551766, sGC-alpha1, LOC409236, LOC102654955, LOC551861, LOC410557, LOC726120, LOC410059, ATP5G2, LOC727483, LOC551527, LOC408432, LOC408868, TyHyd, LOC726463**	$6.23x10^{-04}$
	ATP synthesis coupled electron transport	**LOC725712, LOC412396, LOC725881, LOC100578782, LOC409586, LOC724264, LOC551757, LOC413340, LOC409473,**	$6.98x10^{-03}$

	(GO:0042773)	**LOC726747, LOC408837, LOC726316, Uqcr11, LOC552610, LOC551541**	
	generation of precursor metabolites and energy (GO:0006091)	LOC550767, LOC409773, **LOC550686, LOC408734, LOC552105, LOC411014, LOC107964213, LOC725325, LOC551154, LOC408818, Tpi, LOC725712, LOC410122, LOC102655754, LOC411576, LOC725566, LOC409549, LOC410396, LOC412396, LOC408446, LOC725881, LOC727599, LOC725797, LOC100578782, LOC409586, LOC724264, LOC408950, LOC551757, LOC413340, LOC409473, LOC726747, LOC552128, LOC408837, LOC409292, LOC410059, LOC726316, Uqcr11, LOC552610, LOC551541, CytC**	$9.49x10^{-03}$
Cellular Component	mitochondrion (GO:0005739)	LOC411790, LOC412308, LOC409667, LOC100576847, LOC102655740, LOC410410, LOC410325, LOC725527, LOC408539, LOC551710, **LOC725105, LOC412796, LOC102654379, LOC551523, LOC725240, LOC413879, LOC552526, LOC408352, LOC408734, LOC413228, LOC552635, LOC411304, LOC408569, LOC552644, LOC725854, LOC552802, LOC551337, LOC411142, LOC410856, LOC724988, LOC550915, LOC726498, LOC412082, LOC411351, LOC413438, LOC413774, LOC413762, LOC412409, LOC100577341, LOC413186, LOC552354, LOC725705, LOC408968, LOC413781, LOC411448, LOC727014, LOC724790, LOC551329, LOC412984, LOC408548, LOC551939, LOC102654007, LOC410791, LOC410612, Coq7, LOC726731, LOC411924, LOC726061, LOC551325, LOC725712, LOC100576960, LOC552152, LOC551158, LOC726617, LOC726239, LOC408511, LOC725566, LOC726902, LOC552009, LOC727026, Ndufb2, LOC100302106, LOC412396, LOC408446, Ant, LOC551660, LOC725315, Cox6b1, LOC725881, LOC727599, LOC551042, LOC725797, LOC100578782, LOC409586, LOC724264, Ndufs5, LOC551757, LOC410022, LOC724827, LOC413517, LOC413340, LOC409473, LOC726747, LOC411677, LOC102654955, LOC551861, LOC725253, LOC552128,**	$1.26x10^{-12}$

	**LOC410557, LOC408837, LOC409292, LOC726120, LOC726316, LOC727483, Uqcr11, LOC552610, LOC551541, CytC**	
mitochondrial inner membrane (GO:0005743)	LOC411790, LOC725527, **LOC725105, LOC102654379, LOC408734, LOC408569, LOC551337, LOC726498, LOC412082, LOC412409, LOC413186, LOC725705, LOC408968, LOC413781, LOC408548, LOC102654007, Coq7, LOC725712, LOC725566, LOC726902, LOC552009, Ndufb2, LOC412396, Ant, LOC725881, LOC727599, LOC551042, LOC100578782, LOC724264, LOC551757, LOC413517, LOC413340, LOC409473, LOC726747, LOC411677, LOC102654955, LOC551861, LOC725253, LOC410557, LOC408837, LOC726120, LOC726316, LOC727483, Uqcr11, LOC552610, LOC551541**	$6.40x10^{-07}$
mitochondrial envelope (GO:0005740)	LOC411790, LOC409667, LOC102655740, LOC725527, LOC408539, **LOC725105, LOC102654379, LOC551523, LOC408734, LOC408569, LOC551337, LOC411142, LOC550915, LOC726498, LOC412082, LOC412409, LOC413186, LOC552354, LOC725705, LOC408968, LOC413781, LOC408548, LOC102654007, LOC410791, Coq7, LOC726061, LOC551325, LOC725712, LOC408511, LOC725566, LOC726902, LOC552009, LOC727026, Ndufb2, LOC412396, Ant, LOC725881, LOC727599, LOC551042, LOC100578782, LOC724264, LOC551757, LOC413517, LOC413340, LOC409473, LOC726747, LOC411677, LOC102654955, LOC551861, LOC725253, LOC410557, LOC408837, LOC726120, LOC726316, LOC727483, Uqcr11, LOC552610, LOC551541, CytC**	$6.40x10^{-07}$
organelle inner membrane (GO:0019866)	LOC411790, LOC725527, **LOC725105, LOC102654379, LOC408734, LOC408569, LOC551337, LOC726498, LOC412082, LOC412409, LOC413186, LOC725705, LOC408968, LOC413781, LOC408548, LOC102654007, Coq7, LOC725712, LOC725566, LOC726902, LOC552009, Ndufb2, LOC412396, Ant, LOC725881, LOC727599, LOC551042, LOC100578782, LOC724264, LOC551757, LOC413517, LOC413340, LOC409473, LOC726747, LOC411677, LOC102654955, LOC551861, LOC725253,**	$6.40x10^{-07}$

	**LOC410557, LOC408837, LOC726120, LOC726316, LOC727483, Uqcr11, LOC552610, LOC551541**	
organelle envelope (GO:0031967)	LOC411790, LOC409667, LOC102655740, LOC413675, LOC411918, LOC725527, LOC408539, LOC412464, LOC410343, **LOC725105, LOC102654379, LOC551523, LOC408734, LOC408569, LOC551337, LOC411142, LOC550915, LOC726498, LOC412082, LOC412409, LOC413186, LOC552354, LOC725705, LOC408968, LOC413781, LOC408548, LOC102654007, LOC410791, Coq7, LOC726061, LOC551325, LOC725712, LOC408511, LOC725566, LOC726902, LOC552009, LOC727026, Ndufb2, LOC412396, Ant, LOC725881, LOC727599, LOC551042, LOC100578782, LOC724264, LOC551757, LOC413517, LOC413340, LOC409473, LOC726747, LOC411677, LOC102654955, LOC551861, LOC725253, LOC410557, LOC408837, LOC726120, LOC726316, LOC727483, Uqcr11, LOC552610, LOC551541, CytC**	$3.15x10^{-06}$
envelope (GO:0031975)	LOC411790, LOC409667, LOC102655740, LOC413675, LOC411918, LOC725527, LOC408539, LOC412464, LOC410343, **LOC725105, LOC102654379, LOC551523, LOC408734, LOC408569, LOC551337, LOC411142, LOC550915, LOC726498, LOC412082, LOC412409, LOC413186, LOC552354, LOC725705, LOC408968, LOC413781, LOC408548, LOC102654007, LOC410791, Coq7, LOC726061, LOC551325, LOC725712, LOC408511, LOC725566, LOC726902, LOC552009, LOC727026, Ndufb2, LOC412396, Ant, LOC725881, LOC727599, LOC551042, LOC100578782, LOC724264, LOC551757, LOC413517, LOC413340, LOC409473, LOC726747, LOC411677, LOC102654955, LOC551861, LOC725253, LOC410557, LOC408837, LOC726120, LOC726316, LOC727483, Uqcr11, LOC552610, LOC551541, CytC**	$3.15x10^{-06}$
mitochondrial membrane (GO:0031966)	LOC411790, LOC102655740, LOC725527, LOC408539, **LOC725105, LOC102654379, LOC408734, LOC408569, LOC551337, LOC411142, LOC550915, LOC726498, LOC412082, LOC412409, LOC413186, LOC725705, LOC408968, LOC413781, LOC408548, LOC102654007, LOC410791, Coq7, LOC551325, LOC725712,**	$2.36x10^{-05}$

		**LOC725566, LOC726902, LOC552009, Ndufb2, LOC412396, Ant, LOC725881, LOC727599, LOC551042, LOC100578782, LOC724264, LOC551757, LOC413517, LOC413340, LOC409473, LOC726747, LOC411677, LOC102654955, LOC551861, LOC725253, LOC410557, LOC408837, LOC726120, LOC726316, LOC727483, Uqcr11, LOC552610, LOC551541**	
	cytoplasm (GO:0005737)	Hex110, LOC410994, LOC552313, LOC409435, LOC408987, LOC408650, LOC412544, LOC409520, LOC411378, LOC411147, LOC411790, LOC412308, LOC726887, Cyp6as5, LOC409667, LOC409023, LOC551986, PRF, LOC551466, LOC409576, LOC100576847, LOC550827, LOC725789, LOC551282, LOC102655740, LOC412097, LOC408808, LOC413613, LOC410410, nanos, LOC409485, LOC412823, LOC409313, LOC412608, LOC413742, Arp1, LOC100576667, LOC410325, Ripk5, LOC724947, LOC409809, LOC552560, LOC550716, EF1a-F2, LOC725360, LOC725527, LOC411088, LOC551535, LOC100576432, LOC408388, LOC408539, LOC551420, LOC552363, LOC102653815, LOC406152, LOC410808, LOC408687, LOC409543, LOC408301, LOC726419, LOC724741, LOC551472, LOC551710, LOC411765, LOC552766, LOC410869, LOC551438, LOC551578, LOC550798, LOC409301, LOC411533, LOC411923, LOC411799, LOC551802, LOC412511, LOC413614, LOC412085, Gpdh, LOC552346, LOC411970, LOC412169, LOC409321, LOC725018, LOC413299, LOC410614, LOC551436, LOC725817, LOC411870, LOC551408, LOC413643, LOC725358, LOC552540, LOC408751, LOC551184, LOC550694, LOC409724, LOC726546, LOC551771, **LOC726894, LOC550700, LOC726609, LOC726205, LOC102655898, LOC725105, LOC412119, LOC550975, LOC412796, LOC102654379, LOC552001, LOC409015, LOC102656006, LOC412710, LOC725134, LOC725742, LOC551523, LOC409934, LOC102655877, LOC725240, LOC411654, LOC413879, LOC727300, LOC552533, LOC724188, LOC550686, LOC411459, LOC551477, LOC409487, LOC552526, LOC724496, LOC408352,**	$8.86x10^{-05}$

	**LOC408734, LOC413228, LOC552014, LOC725680, LOC726164, LOC413601, LOC552705, LOC725194, LOC100577378, LOC551580, LOC552635, LOC409055, LOC411304, LOC408569, LOC552644, LOC725854, LOC413889, LOC409905, LOC552802, LOC551337, LOC726176, LOC107964213, LOC100579024, LOC551978, LOC411142, LOC725977, LOC551216, LOC410856, LOC552377, LOC724988, LOC550915, LOC726498, LOC412082, LOC411351, LOC413438, LOC413774, LOC551961, LOC551354, LOC413762, LOC412409, LOC724241, LOC552745, LOC412886, LOC100577341, LOC413186, LOC552519, LOC552354, LOC100578006, LOC725705, LOC408968, LOC413781, LOC411448, LOC552181, LOC727014, LOC724790, Cry2, Syt1, LOC551329, LOC412984, LOC408548, LOC551939, LOC102654007, LOC100576735, LOC410791, LOC410612, Coq7, LOC726731, LOC411924, LOC726061, LOC551325, LOC725712, LOC100576960, LOC413139, LOC409299, LOC410996, LOC552152, LOC551158, LOC726617, LOC726239, LOC408511, LOC102654353, Glob1, LOC102655754, LOC725566, LOC726902, LOC552009, LOC727026, Ndufb2, LOC100302106, LOC412396, LOC408614, LOC408446, Ant, LOC551660, LOC725315, LOC100577548, Cox6b1, LOC725881, LOC727599, LOC551042, TpnI, LOC551631, LOC725797, LOC410371, LOC100578782, LOC409586, Gycbeta1, LOC724264, Ndufs5, LOC551757, LOC410022, LOC724827, Ef-1a-f1, LOC413517, LOC413340, LOC409473, LOC726747, LOC411677, sGC-alpha1, LOC102654955, LOC551861, LOC725253, LOC552128, TpnT, LOC410557, LOC408837, LOC409292, LOC726120, LOC726316, LOC727483, Uqcr11, LOC552610, LOC107964319, LOC551541, CytC**	
inner mitochondrial membrane protein complex (GO:0098800)	**LOC102654379, LOC408569, LOC725712, LOC726902, LOC552009, Ndufb2, LOC412396, LOC551042, LOC100578782, LOC551757, LOC413340, LOC726747, LOC102654955,**	$2.06x10^{-03}$

		**LOC551861, LOC725253, LOC410557, LOC408837, LOC726120, LOC726316, LOC727483, Uqcr11, LOC552610, LOC551541**	
	mitochondrial protein-containing complex (GO:0098798)	LOC410325, **LOC102654379, LOC408569, LOC727014, LOC724790, LOC726731, LOC726061, LOC725712, LOC551158, LOC726902, LOC552009, Ndufb2, LOC412396, LOC551042, LOC100578782, LOC551757, LOC410022, LOC413340, LOC726747, LOC102654955, LOC551861, LOC725253, LOC410557, LOC408837, LOC726120, LOC726316, LOC727483, Uqcr11, LOC552610, LOC551541**	2.77×10^{-03}
Molecular Function	transporter activity (GO:0005215)	Vhdl, LOC725922, nAChRa9, LOC725462, LOC410967, LOC726762, LOC726412, LOC727346, LOC412764, LOC414051, LOC408777, LOC551263, LOC408467, LOC551845, LOC411790, LOC100578810, LOC411955, LOC410578, LOC551250, LOC550918, LOC408634, LOC408478, LOC413259, LOC102655740, Dat, LOC411935, LOC413739, LOC552592, LOC412430, LOC409467, LOC724952, LOC100578207, LOC408828, nAChRb2, LOC408517, LOC551553, LOC725527, LOC551863, LOC551806, LOC408539, LOC552144, LOC408841, LOC410915, LOC410062, LOC726724, LOC411997, LOC412056, LOC412741, LOC552653, LOC552640, LOC551894, LOC411403, LOC410705, LOC552552, LOC408456, **LOC552704, LOC552410, LOC409865, LOC552001, Amih, LOC411295, LOC726423, LOC552720, LOC409074, LOC552744, LOC100577376, LOC411036, LOC726721, LOC409899, LOC408402, LOC409055, LOC413959, LOC551883, LOC409666, LOC411052, LOC552476, LOC409565, LOC409932, LOC551093, LOC412431, LOC551337, LOC412830, LOC408525, LOC552727, nAChRa2, LOC100578899, LOC409192, LOC550915, Para, LOC409636, nAChRb1, LOC725219, LOC410278, LOC551961, nAChRa8, pHCl, LOC409897, LOC409056, nAChRa6, LOC411764, LOC551167, LOC552149, LOC410803, LOC409715, LOC410791, LOC410612, LOC410997, LOC412007, LOC409073, LOC100578218, LOC552792, LOC551325, LOC410805, LOC551454, LOC409924, LOC413816, LOC551782, LOC408769,**	4.03×10^{-06}

		**nAChRa7, LOC410788, Nmdar1, Gat-a, LOC102653800, LOC408992, LOC100578557, LOC409114, LOC409646, LOC412396, GluCl, Amel_8916, Ant, LOC409919, Eaat-2, LOC551660, LOC725315, LOC725881, LOC406124, LOC552699, LOC726793, Gat-1B, LOC410371, LOC552682, LOC411732, LOC408909, LOC724264, LOC551766, LOC724827, LOC551680, LOC413517, LOC726747, LOC412777, LOC409236, LOC102654955, LOC551861, LOC411411, LOC410557, LOC408837, LOC726120, ATP5G2, LOC726316, LCCH3, LOC727483, LOC725346, Uqcr11, LOC412220, LOC552610, Hiscl1, LOC551541, LOC408696, LOC551604, LOC725165, LOC412925, Melt, Vg**	
	transmembrane transporter activity (GO:0022857)	LOC725922, nAChRa9, LOC725462, LOC410967, LOC726762, LOC726412, LOC727346, LOC412764, LOC414051, LOC408777, LOC551263, LOC408467, LOC551845, LOC411790, LOC100578810, LOC410578, LOC550918, LOC408634, LOC408478, LOC413259, LOC102655740, Dat, LOC411935, LOC413739, LOC552592, LOC412430, LOC409467, LOC724952, LOC100578207, LOC408828, nAChRb2, LOC408517, LOC551553, LOC725527, LOC551863, LOC551806, LOC408539, LOC552144, LOC408841, LOC410915, LOC410062, LOC726724, LOC411997, LOC412056, LOC412741, LOC552653, LOC552640, LOC551894, LOC411403, LOC410705, LOC552552, LOC408456, **LOC552704, LOC552410, LOC552001, Amih, LOC411295, LOC726423, LOC552720, LOC409074, LOC552744, LOC100577376, LOC411036, LOC726721, LOC409899, LOC408402, LOC409055, LOC413959, LOC551883, LOC409666, LOC411052, LOC552476, LOC409565, LOC409932, LOC551093, LOC412431, LOC551337, LOC412830, LOC408525, LOC552727, nAChRa2, LOC100578899, LOC550915, Para, nAChRb1, LOC725219, LOC410278, LOC551961, nAChRa8, pHCl, LOC409897, LOC409056, nAChRa6, LOC411764, LOC551167, LOC552149, LOC410803, LOC409715, LOC410791, LOC410612, LOC410997, LOC412007, LOC409073,**	$1.01x10^{-05}$

	**LOC100578218, LOC552792, LOC551325, LOC410805, LOC551454, LOC409924, LOC413816, LOC551782, LOC408769, nAChRa7, LOC410788, Nmdar1, Gat-a, LOC102653800, LOC100578557, LOC409114, LOC409646, LOC412396, GluCl, Amel_8916, Ant, LOC409919, Eaat-2, LOC551660, LOC725315, LOC725881, LOC406124, LOC552699, Gat-1B, LOC410371, LOC552682, LOC411732, LOC408909, LOC724264, LOC551766, LOC724827, LOC551680, LOC413517, LOC726747, LOC412777, LOC409236, LOC102654955, LOC551861, LOC411411, LOC410557, LOC408837, LOC726120, ATP5G2, LOC726316, LCCH3, LOC727483, LOC725346, Uqcr11, LOC412220, LOC552610, Hiscl1, LOC551541, LOC551604, LOC725165, LOC412925, Melt**	
channel activity (GO:0015267)	nAChRa9, LOC727346, LOC408777, LOC408467, LOC551845, LOC413259, LOC100578207, nAChRb2, LOC551806, LOC408841, LOC410915, LOC726724, LOC552640, LOC551894, LOC552552, **Amih, LOC726423, LOC100577376, LOC411036, LOC551883, LOC412830, LOC408525, nAChRa2, LOC100578899, Para, nAChRb1, LOC725219, nAChRa8, pHCl, nAChRa6, LOC411764, LOC552149, LOC552792, LOC551325, LOC410805, LOC551454, LOC551782, nAChRa7, LOC410788, Nmdar1, LOC102653800, LOC409114, GluCl, Amel_8916, LOC406124, LOC552699, LOC552682, LOC411732, LOC551766, LOC551680, LOC409236, LCCH3, LOC727483, Hiscl1, LOC551604, LOC725165, Melt**	1.12×10^{-03}
structural constituent of cuticle (GO:0042302)	CPR21, CPR1, CPR13, LOC102653832, CPR12, LOC102654371, CPR16, **CPR17, CPR11, LOC107964828, LOC726725, CPR27, CPR3, LOC727578, CPR6, CPR5, LOC724624, CPR14, CPR19**	1.12×10^{-03}
proton transmembrane transporter activity (GO:0015078)	LOC725527, **LOC552410, LOC411295, LOC552720, LOC409074, LOC409055, LOC552476, LOC551093, LOC551961, LOC409114, LOC412396, LOC552699, LOC552682, LOC551766, LOC726747, LOC409236, LOC102654955, LOC551861, LOC410557, LOC408837, LOC726120, ATP5G2, LOC726316, LOC727483, Uqcr11, LOC552610, LOC551541**	1.12×10^{-03}

passive transmembrane transporter activity (GO:0022803)	nAChRa9, LOC727346, LOC408777, LOC408467, LOC551845, LOC413259, LOC100578207, nAChRb2, LOC551806, LOC408841, LOC410915, LOC726724, LOC552640, LOC551894, LOC552552, **Amih, LOC726423, LOC100577376, LOC411036, LOC551883, LOC412830, LOC408525, nAChRa2, LOC100578899, Para, nAChRb1, LOC725219, nAChRa8, pHCl, nAChRa6, LOC411764, LOC552149, LOC552792, LOC551325, LOC410805, LOC551454, LOC551782, nAChRa7, LOC410788, Nmdar1, LOC102653800, LOC409114, GluCl, Amel_8916, LOC406124, LOC552699, LOC552682, LOC411732, LOC551766, LOC551680, LOC409236, LCCH3, LOC727483, Hiscl1, LOC551604, LOC725165, Melt**	1.12×10^{-03}
monoatomic ion transmembrane transporter activity (GO:0015075)	nAChRa9, LOC727346, LOC408777, LOC408467, LOC551845, LOC100578207, nAChRb2, LOC725527, LOC408539, LOC408841, LOC726724, LOC551894, LOC411403, LOC552552, **LOC552704, LOC552410, Amih, LOC411295, LOC726423, LOC552720, LOC409074, LOC100577376, LOC411036, LOC409055, LOC551883, LOC552476, LOC551093, LOC412830, LOC408525, nAChRa2, LOC100578899, LOC550915, Para, nAChRb1, LOC725219, LOC551961, nAChRa8, pHCl, nAChRa6, LOC411764, LOC410803, LOC409715, LOC552792, LOC551325, LOC410805, LOC551454, LOC551782, nAChRa7, LOC410788, Nmdar1, LOC102653800, LOC409114, LOC412396, GluCl, Amel_8916, LOC406124, LOC552699, LOC410371, LOC552682, LOC411732, LOC551766, LOC551680, LOC726747, LOC412777, LOC409236, LOC102654955, LOC551861, LOC410557, LOC408837, LOC726120, ATP5G2, LOC726316, LCCH3, LOC727483, Uqcr11, LOC552610, Hiscl1, LOC551541**	2.17×10^{-03}
vitamin B6 binding (GO:0070279)	LOC724239, LOC411796, LOC412460, LOC408509, LOC409721, LOC551593, LOC411447, **LOC410583, LOC413228, LOC410639, LOC411771, LOC410039, LOC552392, LOC409063, LOC410948, LOC411916, LOC410638, LOC410582, LOC409267, LOC725204, LOC412177, LOC408432**	2.53×10^{-03}

	pyridoxal phosphate binding (GO:0030170)	LOC724239, LOC411796, LOC412460, LOC408509, LOC409721, LOC551593, LOC411447, **LOC410583, LOC413228, LOC410639, LOC411771, LOC410039, LOC552392, LOC409063, LOC410948, LOC411916, LOC410638, LOC410582, LOC409267, LOC725204, LOC412177, LOC408432**	2.53x10^{-03}
	inorganic molecular entity transmembrane transporter activity (GO:0015318)	LOC408777, LOC408467, LOC551845, LOC724952, LOC100578207, LOC725527, LOC408841, LOC552640, LOC411403, **LOC552704, LOC552410, Amih, LOC411295, LOC552720, LOC409074, LOC100577376, LOC411036, LOC409055, LOC551883, LOC552476, LOC551093, LOC412830, LOC100578899, Para, LOC725219, LOC551961, pHCl, LOC409056, LOC411764, LOC409715, LOC410805, LOC551454, LOC413816, LOC551782, LOC409114, LOC412396, GluCl, LOC406124, LOC552699, LOC410371, LOC552682, LOC411732, LOC551766, LOC551680, LOC413517, LOC726747, LOC412777, LOC409236, LOC102654955, LOC551861, LOC410557, LOC408837, LOC726120, ATP5G2, LOC726316, LCCH3, LOC727483, Uqcr11, LOC552610, Hiscl1, LOC551541, LOC551604**	3.39x10^{-03}

The table presents the Gene ontology source, term name (term id), associated DEGs, and p-values. DEGs shown in bold are upregulated, while those in normal text are downregulated in spinetoram-treated honeybee adults (STA) compared to spinetoram-treated honeybee larvae (STL). The DEGs in each GO term are displayed from highest downregulation to highest upregulation.

Table S3: List of DEGs for GO categories in STL compared to ATL.

GO source	Term_name (term_id)	Differentially Expressed Genes	P-Value
Molecular Function	pyridoxal phosphate binding (GO:0030170)	LOC410639, LOC724239, LOC410948, LOC410583, **LOC410638**	$2.66x10^{-04}$
	monooxygenase activity (GO:0004497)	Ho, **LOC551179, LOC724175, LOC412209, LOC552418, LOC725159**	$2.66x10^{-04}$
	vitamin B6 binding (GO:0070279)	LOC410639, LOC724239, LOC410948, LOC410583, **LOC410638**	$2.66x10^{-04}$
	tetrapyrrole binding (GO:0046906)	LOC410828, **LOC551179, LOC724175, LOC412209, LOC552418, LOC725159**	$4.29x10^{-04}$
	oxidoreductase activity, acting on paired donors, with incorporation or reduction of molecular oxygen (GO:0016705)	Ho, **LOC551179, LOC724175, LOC412209, LOC552418, LOC725159**	$4.29x10^{-04}$
	heme binding (GO:0020037)	LOC410828, **LOC551179, LOC724175, LOC412209, LOC552418, LOC725159**	$4.29x10^{-04}$
	vitamin binding (GO:0019842)	LOC410639, LOC724239, LOC410948, LOC410583, **LOC410638**	$6.62x10^{-04}$
	iron ion binding (GO:0005506)	**LOC551179, LOC724175, LOC412209, LOC552418, LOC725159**	$2.18x10^{-03}$
	oxidoreductase activity (GO:0016491)	LOC410828, Ho, **LOC409066, LOC551179, LOC724175, LOC552600, LOC412209, LOC411140, LOC552418, LOC725159**	$3.66x10^{-03}$
	lyase activity (GO:0016829)	LOC410639, LOC410948, LOC726262, LOC100577841, **LOC410638**	$3.66x10^{-03}$

The table presents the Gene ontology source, term name (term id), associated DEGs, and p-values. DEGs shown in bold are upregulated, while those in normal text are downregulated in spinetoram-treated honeybee larvae (STL) compared to acetone-treated honeybee larvae (ATA). The DEGs in each GO term are displayed from highest downregulation to highest upregulation.

Table S4: List of DEGs for GO categories in STA compared to ATA.

**GO source**	**Term_name (term_id)**	**Differentially Expressed Genes**	**P-Value**
Biological Process	intracellular sterol transport (GO:0032366)	LOC724386, LOC100577553	$1.57x10^{-02}$
	intracellular lipid transport (GO:0032365)	LOC724386, LOC100577553	$1.57x10^{-02}$
	sterol transport (GO:0015918)	LOC724386, LOC100577553	$1.57x10^{-02}$
	organic hydroxy compound transport (GO:0015850)	LOC724386, LOC100577553	$1.57x10^{-02}$
	lipid transport (GO:0006869)	LOC724386, LOC100577553, **Vhdl**	$4.27x10^{-02}$
	lipid localization (GO:0010876)	LOC724386, LOC100577553, **Vhdl**	$4.33x10^{-02}$

The table presents the Gene ontology source, term name (term id), associated DEGs, and p-values. DEGs shown in bold are upregulated, while those in normal text are downregulated in spinetoram-treated honeybee adults (STA) compared to acetone-treated honeybee adults (ATA). The DEGs in each GO term are displayed from highest downregulation to highest upregulation.

Table S5: List of significant DEGs for each enriched KEGG pathway in ATA compared to ATL.

KEGG Pathway	Differentially Expressed Genes	PValue	Bonferroni	FDR
Oxidative phosphorylation	LOC100576847, LOC551917, LOC725527, LOC410617, **LOC552410, LOC408734, LOC409074, LOC552720, LOC409420, LOC552476, LOC409055, LOC551093, LOC551961, LOC411892, LOC113219091, LOC100578006, LOC412810, LOC725712, LOC727026, Ndufs1, LOC102653862, LOC102654169, LOC100302106, LOC409114, LOC409549, LOC725566, LOC725315, Ndufb2, LOC724719, LOC412396, LOC726297, Cox6b1, LOC409103, LOC551660, LOC552809, LOC552424, LOC725881, LOC551042, LOC408367, LOC727599, LOC413014, LOC552671, LOC724264, LOC727199, LOC725797, LOC409586, LOC409793, LOC408909, LOC552699, LOC551078, LOC551757, LOC551866, LOC724827, LOC726222, LOC551861, Cox6c, LOC551766, LOC726747, LOC413891, Ndufs5, LOC552682, LOC412328, LOC409930, LOC409473, LOC408477, LOC551169, LOC100578782, LOC413340, LOC409236, LOC550667, LOC727049, LOC726042, LOC411411, LOC727483, LOC411183, LOC102654955, LOC726120, LOC726316, LOC410557, LOC408837, ATP5G2, LOC413605, Uqcr11, LOC552610, CytC**	5.59×10^{-61}	7.54×10^{-59}	7.54×10^{-59}

Motor proteins	LOC410994, LOC725469, LOC724193, LOC552508, LOC411018, LOC551711, LOC113218800, LOC726506, LOC413205, LOC551109, LOC725593, LOC552512, LOC412092, LOC725239, LOC409869, LOC411226, LOC412638, LOC550827, LOC100578129, LOC100577649, LOC100578990, LOC724859, LOC726683, Arp1, LOC552070, LOC408388, LOC410960, LOC552766, LOC551438, LOC726456, LOC100576290, LOC410614, **LOC411894, LOC413097, LOC551652, LOC412262, LOC408444, LOC409530, LOC726176, LOC552299, LOC726015, LOC100578704, LOC726044, LOC410851, LOC102656311, LOC724291, LOC411197, LOC410996, LOC413837, LOC100577548, LOC412886, LOC550973, LOC409937, LOC411814, LOC725479, LOC408414, LOC408583, LOC410959, TpnI, LOC410489, TpnT, LOC409843, LOC411316**	4.30×10^{-26}	5.81×10^{-24}	2.90×10^{-24}
Neuroactive ligand-receptor interaction	LOC100576637, LOC100576135, LOC726755, nAChRa9, Akhr, LOC113218562, LOC411760, LOC413829, LOC413164, mGlutR1, LOC724495, LOC552552, **LOC412830, Dop3, LOC410140, LOC412299, LOC100578877, Dop1, LOC411611, LOC412818, 5-HT2alpha, LOC413997, Crzr, 5-HT1, SIFR, LOC411420, LOC726953, 5-HT2beta, DopR2, Nmdar1, LOC113218647, LOC409143, Amel_8916, LOC411220, LOC406124, LOC408534, LOC409626,**	6.09×10^{-23}	8.22×10^{-21}	2.74×10^{-21}

	**LCCH3, LOC726935, LOC410979, LOC410894, LOC552301, LOC113219265**			
Citrate cycle (TCA cycle)	LOC412843, LOC551917, LOC412876, LOC409485, LOC100578735, **Pcl, LOC408734, LOC412522, LOC550686, LOC411014, LOC413768, LOC551403, LOC100576646, LOC410396, LOC551958, LOC409155, LOC408446, LOC409549, LOC725566, LOC551631, LOC724321, LOC551103, LOC408286, LOC408950, LOC551169, LOC550667, LOC552128, LOC409292, LOC410059, PDHB, LOC551039**	$1.61 X 10^{-20}$	$2.17 X 10^{-18}$	$5.43 X 10^{-19}$
Glycolysis / Gluconeogenesis	LOC412843, LOC411188, LOC411140, LOC408559, LOC727456, LOC100578735, LOC725351, LOC412362, LOC413336, LOC409773, **LOC725455, LOC411202, LOC412522, LOC552086, LOC409624, LOC724619, LOC550785, LOC551154, LOC408818, LOC409751, LOC102655754, LOC410122, Tpi, LOC552007, LOC552678, LOC100576646, LOC411576, LOC552736, LOC551927, LOC551631, LOC724724, LOC551103, LOC100577002, LOC551005, PDHB, LOC551039**	$2.87 X 10^{-20}$	$3.88 X 10^{-18}$	$7.76 X 10^{-19}$
Purine metabolism	LOC100577675, LOC412619, LOC552840, LOC552657, LOC725673, LOC724603, LOC551770, LOC412362, LOC409846, LOC724933, LOC552535, LOC414008, LOC410482, LOC550673, LOC409444, LOC412573, **LOC726205, LOC410630, LOC102656439, LOC727293, LOC552583, LOC551523, LOC413601, LOC552514,**	$9.91 X 10^{-20}$	$1.34 X 10^{-17}$	$2.23 X 10^{-18}$

	**LOC726514, LOC551373, LOC726444, LOC410676, LOC411288, LOC724389, LOC726069, LOC724131, LOC408441, Ac3, LOC102653763, LOC552316, LOC410539, LOC409299, LOC413048, LOC552216, LOC408699, Gycbeta1, sGC-alpha1, Adk1**			
ATP-dependent chromatin remodeling	LOC725450, LOC552031, LOC411503, LOC551779, LOC552824, LOC107963990, Brd7, LOC100576266, LOC412046, Arp1, LOC413793, LOC551071, LOC724311, LOC410502, LOC552094, LOC409501, LOC725308, LOC100576104, LOC725795, LOC412077, LOC726816, LOC552200, LOC413181, LOC411727, LOC551881, LOC411279, LOC408891, LOC726619, LOC411629, LOC413318, LOC724678, LOC413341, LOC552582, LOC100578927, LOC412742, LOC410033, **LOC550814, LOC551914, LOC100576784, LOC552481, LOC100578006, LOC551521**	1.85×10^{-19}	2.50×10^{-17}	3.57×10^{-18}
Wnt signaling pathway	LOC413500, LOC410190, LOC551124, LOC724378, LOC413502, LOC411919, LOC552340, LOC552805, LOC100576247, LOC725720, LOC552138, LOC551508, LOC410956, LOC724501, LOC409158, LOC551021, LOC551517, LOC724997, LOC410724, LOC413616, LOC413501, LOC726671, LOC413183, LOC410808, LOC410129, LOC552545, LOC408791, LOC409050, arm, LOC726280, LOC413169, LOC409321, LOC408890, **LOC408278, Pkc, LOC725511, LOC408996, LOC100579005,**	1.56×10^{-17}	2.11×10^{-15}	2.63×10^{-16}

	**LOC409791, LOC107963993, LOC552419, Camkii, LOC724471**			
Pyruvate metabolism	LOC412843, LOC411188, LOC411140, LOC408559, LOC726218, LOC412876, LOC100578735, LOC411813, LOC409773, **LOC411202, LOC552568, LOC412522, LOC552286, LOC409624, LOC411014, LOC409682, LOC552007, LOC410042, LOC552639, LOC100576646, LOC551927, LOC551631, LOC724321, LOC551103, LOC408950, PDHB, LOC551039, LOC724904**	5.25×10^{-15}	7.09×10^{-13}	7.88×10^{-14}
mTOR signaling pathway	LOC413500, LOC551124, LOC413502, LOC411919, LOC100576247, LOC411297, LOC551198, LOC412031, LOC725789, LOC411935, LOC409702, LOC551021, LOC408577, LOC413501, LOC550857, Pten, LOC551512, LOC552381, LOC409050, LOC413430, LOC409725, LOC410201, LOC725647, LOC724472, InR-2, LOC552718, **LOC727552, LOC409317, LOC725584, LOC412531, LOC409036, LOC727159, LOC552720, LOC409577, LOC552476, Pkc, LOC409055, LOC551093, LOC725511, LOC551961, LOC411892, LOC100578725**	1.30×10^{-14}	1.75×10^{-12}	1.75×10^{-13}

The table presents the pathways, associated DEGs, p-values, Bonferroni corrections, and false discovery rates (FDR). DEGs shown in bold are upregulated, while those in normal text are downregulated in acetone-treated honeybee adults (ATA) compared to acetone-treated honeybee larvae (ATL). The DEGs in each KEGG pathway are displayed from highest downregulation to highest upregulation.

Table S6: List of significant DEGs for each enriched KEGG pathway in STA compared to STL.

KEGG Pathway	Differentially Expressed Genes	PValue	Bonferroni	FDR
Oxidative phosphorylation	LOC551917, LOC100576847, LOC725527, LOC410617, **LOC552410, LOC411295, LOC552720, LOC409074, LOC409420, LOC408734, LOC409055, LOC552476, LOC551093, LOC411892, LOC551961, LOC100578006, LOC113219091, LOC412810, LOC725712, Ndufs1, LOC102653862, LOC725566, LOC409549, LOC727026, LOC409114, Ndufb2, LOC102654169, LOC100302106, LOC724719, LOC412396, LOC551660, LOC552424, LOC725315, LOC552671, LOC409103, Cox6b1, LOC725881, LOC727599, LOC551042, LOC552699, LOC726297, LOC725797, LOC408367, LOC552809, LOC409793, LOC551078, LOC100578782, LOC552682, LOC726222, LOC409586, LOC408909, LOC727199, LOC551866, LOC724264, Ndufs5, LOC551757, LOC413014, LOC409930, LOC551766, LOC408477, LOC724827, LOC412328, LOC413340, LOC413891, LOC409473, LOC726747, LOC551169, LOC409236, LOC102654955, LOC551861, LOC727049, LOC411411, Cox6c, LOC410557, LOC408837, LOC726042, LOC411183, LOC550667, LOC726120, ATP5G2, LOC726316, LOC727483, LOC413605, Uqcr11, LOC552610, CytC**	1.94×10^{-62}	2.60×10^{-60}	2.60×10^{-60}

Motor proteins	LOC725469, LOC410994, LOC552508, LOC724193, LOC551711, LOC411018, LOC551109, LOC113218800, LOC726506, LOC552512, LOC413205, LOC725593, LOC412092, LOC100578129, LOC411226, LOC550827, LOC409869, LOC412638, LOC100577649, LOC724859, LOC552070, LOC725239, LOC102655879, Arp1, LOC410960, LOC726683, LOC408388, LOC100578990, LOC552766, LOC551438, LOC100576290, LOC410614, LOC725068, LOC726456, LOC412267, **LOC412174, LOC551652, LOC408444, LOC552299, LOC726176, LOC102656311, LOC410851, LOC726044, LOC100578704, LOC413097, LOC726015, LOC412886, LOC724291, LOC413837, LOC410996, LOC411197, LOC409937, LOC550973, LOC408414, LOC408583, LOC725479, LOC410489, LOC410959, LOC100577548, TpnI, LOC411814, TpnT, LOC409843, LOC411316**	7.58×10^{-27}	1.02×10^{-24}	5.08×10^{-25}
Purine metabolism	LOC100577675, LOC412467, LOC412619, LOC552840, LOC551448, LOC412362, LOC724603, LOC725673, LOC409444, LOC551770, LOC552657, LOC409846, LOC414008, LOC724933, LOC552535, LOC412573, LOC410482, **LOC726205, LOC551523, LOC727534, LOC102656439, LOC727293, LOC552514, LOC726514, LOC413601, LOC551373, LOC410630, LOC726262, LOC726444, LOC724389, LOC410676, LOC411288, LOC410539,**	1.29×10^{-21}	1.73×10^{-19}	5.77×10^{-20}

	**LOC726069, LOC408441, LOC102653763, LOC724131, LOC552316, LOC409299, Ac3, LOC413048, LOC552216, LOC408699, Gycbeta1, sGC-alpha1, Adk1**			
Neuroactive ligand-receptor interaction	LOC100576637, LOC100576135, LOC726755, nAChRa9, Akhr, LOC411760, LOC113218562, LOC413829, LOC413164, LOC724473, LOC552552, **LOC726535, LOC100578877, LOC410140, Crzr, Dop3, LOC412299, LOC412830, LOC552761, SIFR, LOC413997, LOC412818, LOC411420, LOC411611, 5-HT2alpha, 5-HT1, Dop1, DopR2, LOC726953, LOC409143, LOC552301, Nmdar1, LOC113218647, LOC410894, Amel_8916, LOC411220, LOC406124, 5-HT2beta, LOC726935, LCCH3, LOC410979**	1.47×10^{-20}	1.97×10^{-18}	4.93×10^{-19}
ATP-dependent chromatin remodeling	LOC725450, LOC552031, LOC726468, LOC411503, LOC551779, Arp1, LOC413793, LOC552824, LOC412046, LOC551071, LOC552094, LOC100576266, LOC107963990, Brd7, LOC724311, LOC410502, LOC413181, LOC409501, LOC100576104, LOC725308, LOC726816, LOC725795, LOC411279, LOC552200, LOC724519, LOC411727, LOC410033, LOC412077, LOC726619, LOC412742, LOC411629, LOC552582, LOC413341, LOC551881, LOC724678, LOC100578927, LOC413318, **LOC550814, LOC551914, LOC100576784, LOC552481, LOC100578006, LOC551521**	2.34×10^{-20}	3.13×10^{-18}	6.27×10^{-19}

Citrate cycle (TCA cycle)	LOC412843, LOC551917, LOC412876, LOC409485, LOC100578735, **LOC550686, LOC408734, LOC412522, LOC411014, LOC413768, LOC551403, LOC100576646, LOC725566, LOC409549, LOC551958, LOC410396, LOC409155, LOC408446, LOC551631, LOC551103, LOC408286, LOC724321, LOC408950, LOC551169, LOC552128, LOC409292, LOC550667, LOC410059, PDHB, LOC551039**	5.38×10^{-19}	7.21×10^{-17}	1.11×10^{-17}
Glycolysis / Gluconeogenesis	LOC408559, LOC411140, LOC411188, LOC412843, LOC727456, LOC725351, LOC412362, LOC100578735, LOC550687, LOC413336, LOC409773, **LOC411189, LOC725455, LOC412522, LOC552086, LOC409624, LOC550785, LOC551154, LOC408818, Tpi, LOC410122, LOC552678, LOC552007, LOC102655754, LOC411576, LOC100576646, LOC552736, LOC551927, LOC724724, LOC551631, LOC551103, LOC100577002, LOC551005, PDHB, LOC551039**	5.79×10^{-19}	7.76×10^{-17}	1.11×10^{-17}
Wnt signaling pathway	LOC413502, LOC724378, LOC411919, LOC413500, LOC551124, LOC552340, LOC410190, LOC552805, LOC552138, LOC100576247, LOC551508, LOC726671, LOC724501, LOC410956, LOC551517, LOC724997, LOC409158, LOC726113, LOC413501, LOC413183, LOC552374, LOC408791, LOC551021, LOC410808, LOC410724, LOC413616, LOC410129, LOC409050, LOC552545, LOC726280, LOC409321, arm, LOC409001, LOC408890,	2.07×10^{-17}	2.78×10^{-15}	3.47×10^{-16}

	**LOC725511, Pkc, LOC100579005, LOC409791, LOC408996, LOC107963993, LOC552419, Camkii, LOC724471**			
Nucleocytoplasmic transport	LOC410447, LOC552567, LOC413018, LOC725332, LOC411865, LOC412817, LOC413344, LOC413675, LOC100576146, LOC727561, LOC411918, LOC413636, LOC410280, LOC100577501, EF1a-F2, LOC413146, LOC409229, LOC725396, LOC412584, LOC409543, LOC412464, LOC411909, LOC413173, LOC410343, LOC413343, LOC726354, LOC409308, LOC408842, LOC550834, LOC412511, LOC100578879, LOC408625, LOC726159, LOC551707, LOC726339, LOC100576125, LOC413365, LOC412835, LOC412721, LOC409672, LOC409866, **LOC725134, LOC412950, LOC413378, LOC102656258, Ef-1a-f1**	5.07 X 10^{-17}	6.80 X 10^{-15}	7.55 X 10^{-16}
mTOR signaling pathway	LOC413502, LOC411919, LOC413500, LOC551124, LOC725789, LOC100576247, LOC410201, LOC411297, LOC411935, LOC551198, LOC551512, LOC409702, LOC412031, Pten, LOC408577, LOC413501, LOC550857, LOC552374, LOC551021, LOC409050, LOC552381, LOC551980, LOC409925, LOC413430, LOC413365, InR-2, LOC724472, LOC552718, LOC724408, LOC409725, **LOC412531, LOC409036, LOC411295, LOC727159, LOC552720, LOC409055, LOC725511, LOC552476, LOC409577,**	4.09 X 10^{-16}	5.48 X 10^{-14}	5.48 X 10^{-15}

	**LOC551093, Pkc, LOC411892, LOC551961, LOC100578725**			

The table presents the pathways, associated DEGs, p-values, Bonferroni corrections, and false discovery rates (FDR). DEGs shown in bold are upregulated, while those in normal text are downregulated in spinetoram-treated honeybee adults (STA) compared to spinetoram-treated honeybee larvae (STL). The DEGs in each KEGG pathway are displayed from highest downregulation to highest upregulation.

Table S7: List of significant DEGs for each enriched KEGG pathway in STL compared to ATL

KEGG Pathway	Differentially Expressed Genes	PValue	Bonferroni	FDR
Tryptophan metabolism	LOC410639, LOC724239, LOC410828, **LOC410638, LOC411140, LOC408559**	3.22×10^{-08}	1.68×10^{-06}	1.68×10^{-06}
Arginine and proline metabolism	LOC410583, **LOC552556, LOC411140, LOC408559**	1.70×10^{-04}	8.82×10^{-03}	4.41×10^{-03}
Motor proteins	LOC411814, LOC410323, LOC411602, LOC113218800, LOC100578990	1.24×10^{-03}	6.46×10^{-02}	2.15×10^{-02}
Tyrosine metabolism	LOC410639, LOC410948, **LOC410638**	1.66×10^{-03}	8.61×10^{-02}	2.15×10^{-02}
Insect hormone biosynthesis	**LOC551179, LOC408559, LOC724216**	2.46×10^{-03}	1.28×10^{-01}	2.55×10^{-02}
Ascorbate and aldarate metabolism	LOC725106, **LOC411140, LOC408559**	3.40×10^{-03}	1.77×10^{-01}	2.95×10^{-02}
Lysosome	**LOC409709, LOC411657, LOC551863, LOC411353**	5.68×10^{-03}	2.95×10^{-01}	4.22×10^{-02}
Lysine degradation	LOC724239, **LOC411140, LOC408559**	1.08×10^{-02}	5.61×10^{-01}	7.01×10^{-02}
Glycolysis / Gluconeogenesis	**LOC411140, LOC408559, LOC724619**	1.87×10^{-02}	9.72×10^{-01}	1.08×10^{-01}
Histidine metabolism	**LOC411140, LOC408559**	2.60×10^{-02}	1.00	1.31×10^{-01}

The table presents the pathways, associated DEGs, p-values, Bonferroni corrections, and false discovery rates (FDR). DEGs shown in bold are upregulated, while those in normal text are downregulated in spinetoram-treated honeybee larvae compared to acetone-treated honeybee larvae. The DEGs in each KEGG pathway are displayed from highest downregulation to highest upregulation.

Table S8. List of oligo primers used for qPCR

Gene Symbol (Sequence ID)	Orientation	Oligo sequence (3' → 5')	Tm (℃)	Product length (bp)
Rpl 13a (XM_623810.5)	Forward	TGGCCATTTACTTGGTCGTT	58.9	191
	Reverse	GAGCACGGAAATGAAATGG	56.5	

ATL vs ATA

Gene Symbol (Sequence ID)	Orientation	Oligo sequence (3' → 5')	Tm (℃)	Product length (bp)
TpnCIIIb (NM_001011652.2)	Forward	CCATTCGGATCCGGGGAAAT	64.1	76
	Reverse	CTGTGTCCGTGTCCTCTTCC	64.0	
Melt (NM_001011607.2)	Forward	TTACATCTATGCGGCCCCTG	61.8	86
	Reverse	CAATTCCCGCTTCCGGATCT	63.2	
LOC725364 (XM_016912430.2)	Forward	GTGGAGTGGAGTGGCTATCG	63.0	95
	Reverse	ATATCCCATGGCGCTGGTTC	62.3	
Hex110 (NM_001101023.1)	Forward	ACAACAAGCAGGACAACAGG	60.1	118
	Reverse	ACCAAGTCCGTTAGAAAGACGA	60.4	
Fibroin4 (NM_001136209.1)	Forward	GATCCCATCCATACTCGCGG	63.4	72
	Reverse	GTCTCCACCTCTTCCCTTGC	64.3	
Vhdl (NM_001331117.1)	Forward	AACGCCTCAATCAGAACGGT	61.2	117
	Reverse	GGATCTACCGAGGTGGCAAG	63.6	

ATL vs STL

Gene Symbol (Sequence ID)	Orientation	Oligo sequence (3' → 5')	Tm (℃)	Product length (bp)

Cpr21 (NM_001270)	Forward	TTGATTTGCGCTGTCTTCGC	59.8	111
	Reverse	GCCATCTACGTTCGGTCCAT	61.9	
Fibroin2 (NM_001136207.1)	Forward	GGCCAAAAACACAGAGGCTG	62.0	89
	Reverse	CGCTGGCTTTAATCGCTTCC	60.8	
Hex110 (NM_001101023.1)	Forward	ACAACAAGCAGGACAACAGGA	61.3	85
	Reverse	ACGTGCCAAAAGTTGTTGATGA	59.1	
LOC100578614 (XM_003251222.4)	Forward	TACAAGCTAGTGGTGCAGGC	61.0	89
	Reverse	TCTCGTTCCACGTCCCAAAG	63.2	
LOC724239 (XM_001120032.5)	Forward	GGAGAGCCGCGACATCATAA	61.6	78
	Reverse	CCAGGTTCGAGTATGGCCTC	63.6	
Mrjp3 (NM_001011601.1)	Forward	GATGTGGACAGATGGCGTGA	62.4	106
	Reverse	GAGGTCCACCTTTGCCCTTT	63.8	

STL vs STA

Gene Symbol (Sequence ID)	Orientation	Oligo sequence (3’ → 5’)	Tm (°C)	Product length (bp)
Melt (NM_001011607.2)	Forward	TACATCTATGCGGCCCCTGA	63.0	93
	Reverse	AACTGCTCCAATTCCCGCTT	61.6	
Obp17 (NM_001040207.1)	Forward	TCTGCTATTTGCGTTTGCGT	58.0	59
	Reverse	GCAGTGCCAATTTCTTTCATGC	58.8	
TpnCIIIa (NM_001318482.1)	Forward	GACAAATTGACGCCGCAAGA	60.3	82
	Reverse	ATTCGTCGAAGTCGAGCGTT	60.4	
Fibroin3 (NM_001136208.1)	Forward	TAGCCAGCGACTTGCAGAAA	60.5	97
	Reverse	CTCCATTTCGTTCGTTCCGC	61.6	
Hex110 (NM_001101023.1)	Forward	ACAACAAGCAGGACAACAGGA	61.3	85
	Reverse	ACGTGCCAAAAGTTGTTGATGA	59.1	

Vhdl (NM_001331117.1)	Forward	TAGTGCGGACAAAGAGCGTT	60.4	76
	Reverse	GGAACCTTCACATGCATCGC	61.3	

ATA vs STA

Gene Symbol (Sequence ID)	Orientation	Oligo sequence (3’ → 5’)	Tm (℃)	Product length (bp)
LOC100576179 (XM_016914040.2)	Forward	CAACGAGACGAAGAGGGTGT	62.3	92
	Reverse	CGTCGAAGATCGCGTACAGA	60.9	
LOC100576555 (XM_003249623.4)	Forward	TGCACACCATCTTTTGCACG	60.3	82
	Reverse	GTCAAAACACTGTCACCGGC	61.7	
LOC724654 (XM_006569927.3)	Forward	AAGTTGCGACACGAGATGGA	60.9	106
	Reverse	TCTGTCCCACCAGGATGCTT	64.4	
LOC724158 (XM_001119914.5)	Forward	AGCTTATTTGAAACCATTGCCAGA	58.4	70
	Reverse	ATGTGCAGTTTCATGTGTTTGA	56.3	
LOC100577533 (XM_026444872.1)	Forward	TTTACGAAACTTTCTGGGAAAAACA	57.3	70
	Reverse	ACATGTCCAATCAAAATACGGCG	59.5	
LOC100576514 (XM_016912033.2)	Forward	TACAAGCTAGTGGTGCAGGC	61.0	89
	Reverse	TCTCGTTCCACGTCCCAAAG	63.2	

STA, spinetoram-treated honeybee larvae; STA, spinetoram-treated honeybee adults; ATL, acetone-treated honeybee larvae; ATA, acetone-treated honeybee adults
